# Plasmonic photothermal release of docetaxel by gold nanoparticles incorporated onto halloysite nanotubes with conjugated 2D8-E3 antibodies for selective cancer therapy

**DOI:** 10.1186/s12951-021-00982-6

**Published:** 2021-08-11

**Authors:** Reza Taheri-Ledari, Wenjie Zhang, Maral Radmanesh, Nicole Cathcart, Ali Maleki, Vladimir Kitaev

**Affiliations:** 1 Catalysts and Organic Synthesis Research Laboratory, Department of Chemistry, Iran University of Science and Technology, Tehran, 16846-13114 Iran; 2 Department of Nuclear Medicine, West China Hospital, Sichuan University, No. 37, Guoxue Alley, Chengdu, 610041 Sichuan People’s Republic of China; 3 Department of Chemistry and Biochemistry, Wilfrid Laurier University, 75 University Ave. W., Waterloo, ON Canada

**Keywords:** Antibody–drug conjugate, Therapeutic nanocomposite, Gold nanoparticles, Plasmonic photothermal drug release, Targeted drug delivery, Ovarian tumor targeting

## Abstract

**Background:**

Applied nanomaterials in targeted drug delivery have received increased attention due to tangible advantages, including enhanced cell adhesion and internalization, controlled targeted release, convenient detection in the body, enhanced biodegradation, etc. Furthermore, conjugation of the biologically active ingredients with the drug-containing nanocarriers (nanobioconjugates) has realized impressive opportunities in targeted therapy. Among diverse nanostructures, halloysite nanotubes (NHTs) with a rolled multilayer structure offer great possibilities for drug encapsulation and controlled release. The presence of a strong hydrogen bond network between the rolled HNT layers enables the controlled release of the encapsulated drug molecules through the modulation of hydrogen bonding either in acidic conditions or at higher temperatures. The latter can be conveniently achieved through the photothermal effect via the incorporation of plasmonic nanoparticles.

**Results:**

The developed nanotherapeutic integrated natural halloysite nanotubes (HNTs) as a carrier; gold nanoparticles (AuNPs) for selective release; docetaxel (DTX) as a cytotoxic anticancer agent; human IgG1 sortilin 2D8-E3 monoclonal antibody (SORT) for selective targeting; and 3-chloropropyltrimethoxysilane as a linker for antibody attachment that also enhances the hydrophobicity of DTX@HNT/Au-SORT and minimizes DTX leaching in body’s internal environment. HNTs efficiently store DTX at room temperature and release it at higher temperatures via disruption of interlayer hydrogen bonding. The role of the physical expansion and disruption of the interlayer hydrogen bonding in HNTs for the controlled DTX release has been studied by dynamic light scattering (DLS), electron microscopy (EM), and differential scanning calorimetry (DSC) at different pH conditions. HNT interlayer bond disruption has been confirmed to take place at a much lower temperature (44 °C) at low pH vs. 88 °C, at neutral pH thus enabling the effective drug release by DTX@HNT/Au-SORT through plasmonic photothermal therapy (PPTT) by light interaction with localized plasmon resonance (LSPR) of AuNPs incorporated into the HNT pores.

**Conclusions:**

Selective ovarian tumor targeting was accomplished, demonstrating practical efficiency of the designed nanocomposite therapeutic, DTX@HNT/Au-SORT. The antitumor activity of DTX@HNT/Au-SORT (apoptosis of 90 ± 0.3%) was confirmed by in vitro experiments using a caov-4 (ATCC HTB76) cell line (sortilin expression > 70%) that was successfully targeted by the sortilin 2D8-E3 mAb, tagged on the DTX@HNT/Au.

**Graphic abstract:**

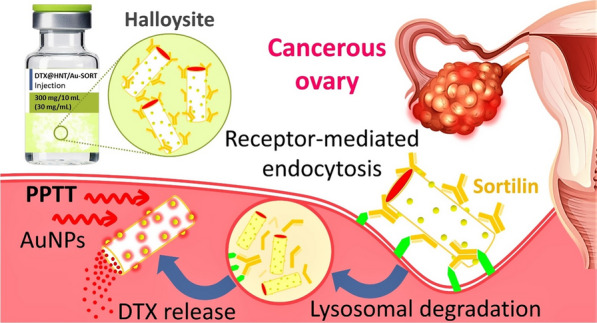

**Supplementary Information:**

The online version contains supplementary material available at 10.1186/s12951-021-00982-6.

## Introduction

Traditional cancer therapy relies upon the administration of cytotoxic chemical agents resulting in multiple negative side effects of chemotherapy due to the susceptibility of normal cells to this treatment [1, 2]. Current strategies to minimize these side effects involve different approaches for targeted drug delivery to specific tissues [3–5], including magnetically driven transport of therapeutic agents [6–8], and/or incorporation of the specific biomolecules, such as aptamers [9], peptide sequences [10], and antibodies [11] onto the surface of the nanoscale carriers for selective delivery of the anti-cancer medication [12, 13]. There are two main types of cancer cells to target: (1) cancer stem cells (CSCs) that could develop into different cell types [14], and (2) differentiated cancer cells (DCCs) that have matured into a particular form of cancer [15]. It is crucial to be able to treat CSCs in order to eradicate the cancer cells at their origin to suppress DCCs. CSC targeting has been actively explored in the recent decade using conjugation of anti-cancer drugs to specific antibodies [16, 17] and other biologically active molecules, such as folic acid, peptides, etc. [18–20]. These biologically active molecules then become responsible for binding with their receptors on the surface of the targeted cells [21, 22]. The strong affinity of antibodies to the corresponding antigens on the cell membrane enables attachment and internalization through endocytosis; as a consequence of the selectivity of the drug delivery, the negative side effects of the administration of the cytotoxic agents is greatly reduced [23]. In this work, we selected a human IgG1 sortilin 2D8-E3 monoclonal antibody (SORT) to target the caov-4 ovary tumor cells due to its good expression (above 70%) by caov-4 cells. In antibody–drug conjugates (ADCs), a cytotoxic agent is attached using a linker [24]. Functionalizing nanoparticles with biologically active receptors is an additional strategy for selective tumor targeting with several successful nanoscale drug delivery systems reported [25–28].

Among diverse heterogeneous nanoscale materials for drug delivery [29, 30], HNTs have attracted significant attention because of their natural tubular layered morphology reminiscent of a rolled carpet, which is advantageous for interlayer drug encapsulation [31]. This HNT architecture is particularly suitable for drug delivery in two aspects: (1) large interior volumes are available both inside the tubes and between the layers, and (2) drug release can be selectively triggered, e.g. by heating or changing pH [31, 32]. In many pharmaceutical products, enteric coating is commonly employed for controlling the release process of the target molecules [33, 34]; this can be accomplished with HNTs as their multilayered structure enables extended or sustained release of the incorporated drug molecules [35–38]. For instance, to overcome the acidic degradation of rabeprazole in the stomach, HNTs have been used for sustained release and to enhance bioavailability by Yurtdas and Yazan [39]. Bio-functionalization of HNTs has resulted in a new class of nano-carriers that can be used for the controlled delivery of drug molecules to treat tumors. HNTs have long-term stability and are non-toxic, making them a promising material for biological applications [40]. Multilayered tubular nanostructures provide a large interior space that can be used for drug encapsulation [39, 40]. Also, tubular cross sections at the ends are reported to enhance cell penetration and cellular uptake [41, 42]. Accumulation of the tubular-shaped nanostructures causes less steric hindrance at the end of tubes, and as a result, their cellular uptake is higher compared to spherical nanostructures [42]. In particular, F-actin protein structures interact strongly with rod- or tubular-shaped particles. These interactions are responsible for the formation of coherent arrangements of actin filaments and rod-like nanostructures inside the cells greatly enhancing the penetration of tumors [43]. In another important study, the efficiency of single-wall carbon nanotubes has been tested with the living samples, and it was found that the nanotube accumulation in tumors is among the highest that can be achieved with nanomaterials [44]. In fact, it has been reported that the tubular shape enables a polyvalency effect, enhances tumor binding affinity, and may also facilitate leaking of nanotubes out of blood micro-vessels to reach tumor cells through vascular and interstitial barriers [44].

The surface chemistry of HNTs features multiple aluminol and silanol groups that can serve as sites for the covalent attachment of different chemical moieties. These HNT hydroxyl groups can also facilitate hydrogen bonding with biomolecules [45]. Previously, it has been reported that ca. 92% of HNT-encapsulated anticancer drug camptothecin can be released in acidic conditions (pH = 5.0) while at neutral pH values, the drug is retained in HNTs enabling selective delivery and release [46]. At low temperatures, silica and alumina networks inside the HNTs are tightly linked together by hydrogen bonding, which enables the encapsulation of drug molecules between the layers. Upon heating, hydrogen bonding between the layers is disrupted so that the rolled layers of HNTs are expanded, and the drug molecules become released [47]. Another useful feature of HNTs is that they can be well dispersed as individual particles and remain stable for months in this colloidal form and are therefore beneficial for drug storage [48]. The mesoporous outer surface of HNTs (pores/cavities of 2 to 50 nm) is also amenable to the incorporation of other nanoscale materials such as metal and metal oxide nanoparticles [49, 50]. This incorporation presents an opportunity for the drug release, e.g. by disruption of HNT interlayer hydrogen bonding and embedding plasmonic AuNPs to absorb light and to trigger heating via Plasmonic PhotoThermal Therapy (PPTT) [51]. Finally, HNTs are advantageous for biomedical applications owing to their biocompatibility and nontoxicity that have been confirmed by toxicity assays with different human cell lines (e.g., epithelial adenocarcinoma cells and dermal fibroblasts) [48–52].

Gold nanoparticles (AuNPs) feature several advantages in cancer therapy: (1) plasmonic photothermal therapy (PPTT) [53] (2) therapeutic enhancement of apoptosis and metastasis of cancer cells [54, 55] and (3) monitoring the targeted drug delivery process through the detection of the AuNPs via computed tomography (CT) imaging [56]. PPTT is accomplished through the exposure of AuNPs to the visible or near-IR light at their localized surface plasmon resonance (LSPR) wavelength that can heat AuNPs via strong interactions of delocalized electrons resulting in high absorption cross-sections [57]. LSPR of AuNPs is advantageously tunable via NP size and morphology [58]. In this context, AuNPs can be successfully used for the controlled release of the entrapped drugs [59], including temperature-responsive polymer gels [60–62].

Bringing together all the advantages of the individual components discussed above, herein, we report DTX@HNT/Au-SORT nanotherapeutic comprised of HNTs with a conjugated monoclonal antibody (mAb) as a biologically active moiety for targeted drug delivery and small (10 nm) plasmonically active AuNPs incorporated into the pores of the HNTs. DTX was selected as a cytotoxic anti-cancer agent, with a mouse IgG1 model monoclonal antibody (sortilin 2D8-E3) that is encoded by a human SORT1 (NT3) receptor used for modification of the HNTs surfaces for DTX encapsulation and to target the caov-4 ovary cancerous cells (> 70% expression). Strong binding of DTX to HNTs is confirmed by DTX loading at a lower temperature, with low drug leaching observed at 37 °C. The designed drug delivery system was demonstrated to be capable of releasing the encapsulated DTX controllably through the LSPR heating of AuNPs under light irradiation. AuNPs serve as hot spots on the outer surface of HNTs that cause the rolled structure of HNTs to expand and to release DTX with the synergy observed at low pH common to tumor cells. As a result, in vitro bioassay experiments performed using 3T3 human normal (fibroblast) and caov-4 (ATCC HTB76) human ovarian cancer cell lines demonstrated high selectivity of DTX@HNT/Au-SORT in cell adhesion and internalization with the notable cell killing potency of ca. 90% at 50 μg/mL of DTX@HNT/Au-SORT under irradiation with white LED light (7 W).

## Experimental

### Reagent and materials

All chemical and biological reagents and instrumentation used in this work are summarized in Tables 1 and 2, respectively.

**Table 1:** Chemical and biological reagents used in this work

Reagent	Brand & purity
3T3 and caov-4 cell lines	ATCC
(3-Chloropropyl)trimethoxysilane (CPTMS)	Sigma-Aldrich, ≥ 97.0%
Crystal violet (tris(4-(dimethylamino)phenyl)methylium chloride)	Merck, ≥ 90.0%
Docetaxel (DTX)	Sigma-Aldrich, purum, ≥ 97.0% (HPLC)
Dulbecco’s modified Eagle’s medium (DMEM)	Sigma-Aldrich
Ethylene glycol	Sigma-Aldrich, spectrophotometric grade, ≥ 99%
Halloysite nanotubes (HNTs) clay	Sigma-Aldrich
Lugol’s solution (iodine 5%, and potassium iodide 10% w/v)	Merck
MTT (3-(4,5-dimethylthiazol-2-yl)-2,5-diphenyl tetrazolium bromide)	Sigma-Aldrich, 98.0%
Paper filter	Whatman (602 h, particle retention < 2 µm)
Deionized water	Merck
Ethanol	Merck, 96%, (synthesis grade)
Toluene	Merck, (chromatographic grade)
Dimethyl sulfoxide (DMSO)	Merck, (analysis grade)
Sortilin 2D8-E3 mAb (SORT)	Avicenna Research Institute, Iran
Sodium borohydride	Sigma-Aldrich, 98.0%
Tetrachloroauric(III) acid trihydrate	Sigma-Aldrich, Supelco, 99%
Trisodium citrate dihydrate	Sigma-Aldrich, ≥ 99.0%

**Table 2:** Research instrumentation used in this work

Instrument	Brand
FTIR^a^ spectroscopy	Shimadzu FTIR-8400S
EDX^b^ spectroscopy	VEGA-TESCAN-XMU
FESEM^c^	Hitachi S-5200
TEM^d^	Philips CM200
TGA^e^	TGA-Bahr-STA 504, under argon
DLS^f^	Horiba (SZ-100)
DSC^g^	Mettler DSC 822/400
ELISA^h^	Bio-Tek ELx800
BET^i^	ASAP 2020 V3.03 E, Micromeritics
Flow cytometry	Agilent
UV–vis spectroscopy	Beckman DU640
Confocal microscopy	Zeiss LMS 700
Ball-milling	Amin Asia Fanavar Pars Co. (IRAN)
White LED light	SABA (7 W)
Thermometer	Fluke (572–2 infrared)
Ultrasound probe	Hielscher (UP100H)

^a^Fourier transform infrared

^b^Energy-dispersive X-ray

^c^Field-emission scanning electron microscopy

^d^Transmission-electron microscopy

^e^Thermogravimetric analysis

^f^Dynamic-light scattering

^g^Differential scanning calorimetry

^h^Enzyme-linked immunosorbent assay

^i^Brunauer–Emmett–Teller

### Synthesis

#### HNT pretreatment protocol

HNTs were first treated by ball-milling (Amin Asia Fanavar Pars Co., Iran) [63] to mechanically grind and separate HNTs. The ball mill uses two vertically installed zirconia grinding bowls with zirconia balls of 0.1 mm in diameter. In a typical grinding procedure, a batch of HNTs (10.0 g) was placed into the mill bowl with 15 grinding balls and grinded using the frequency of 200 Hz for 2 h, with a 5-min break every 15 min. The ground HNT powder was separated from the zirconia balls via ultrasonication (50 kHz, 200 W/L) in deionized water at room temperature for 10 min. The quality of the milling step has been monitored by dynamic-light scattering (DLS), as presented in Fig. 4 and discussion of these data later in the text. Subsequently, HNTs were rinsed three times with a mixture of deionized water and ethanol (1:1 volume ratio, ca. 10 g of HNTs per 120 mL), using centrifugation (11,200 RCF, 15 min) and redispersion between rinses, with the supernatant and heavy aggregates discarded. In the next step, HNT calcination was carried out at 650 °C for 2 h [64]. Subsequently, dry freshly calcined HNTs (2.0 g) were dispersed in 50 mL of hydrochloric acid (HCl, 1 M), via ultrasonication (150 kHz, 200 W/L) at room temperature [65]. Well-dispersed HNTs were then subjected to reflux for 24 h. After the reflux, the mixture was cooled down to room temperature, and activated HNTs were separated via centrifugation (11,200 RCF, 15 min), washed three times with acetone, and dried. In order to improve the uniformity of the size distribution of HNTs after grinding and acid treatment, HNTs were first centrifuged and then treated by dialysis. Specifically, centrifugation of the aqueous dispersions of HNTs (50 mg/mL) was first performed for 40 min at 1200 RCF in a plastic centrifuge tube (Falcon, 50 mL, conical). Then, the heavy sediments were separated by decantation and discarded. The resulting supernatant was ultrasonicated (50 kHz, 200 W/L) for 5 min at room temperature to disperse the agglomerated particles, and centrifugation was repeated using the same 1200 RCF for 2 min. Subsequently, the obtained HNT dispersion (5.0 mL, 50 mg/mL) was transferred into a dialysis bag (100 KDa, Spectra/Por Biotech) and the medium was stirred for the first 24 h in 200 mL PBS (pH = 6.8, 0.1 M). Then, the medium was replaced and the dialysis was continued for additional 48 h in fresh medium. Finally, the dialyzed HNT dispersions were washed with deionized water and dried in a vacuum oven at 50 °C for 24 h.

#### Preparation of HNTs/CPS

In a round bottom flask (100 mL), activated dry HNTs (0.5 g) prepared as described above were weighed and dispersed in ethanol (10 mL) via ultrasonication (50 kHz, 200 W/L) for 15 min. To the same flask, a solution of (3-chloropropyl)trimethoxysilane (CPTMS) in toluene (20% v/v, 10 mL) was added drop by drop, and the mixture was vigorously stirred under reflux conditions for 24 h. Finally, chloropropylsilane-modified HNTs (HNTs/CPS) were collected via centrifugation (11,200 RCF, 15 min) and redispersed again in ethanol (10 mL) using ultrasonication. The precipitation-redispersion cycles were repeated three times to remove unreacted CPTMS.

#### Preparation of DTX@HNTs/CPS

In a glass test tube (13 by 100 mm, equipped with a threaded cap), 0.100 g of HNTs/CPS was dispersed in 5.0 mL of an equivolume mixture of ethanol and deionized water by ultrasonication until a stable suspension was obtained. In the next step, DTX solution (5.0 mL, 0.1 M in ethanol) was added, and the contents of the glass tube were continuously stirred for 12 h in an orbital shaker at 120 rpm, being protected from light by wrapping a test tube with an aluminum foil. Subsequently, centrifugation was performed (11,200 RCF, 15 min) to separate a light yellow precipitate. To remove the weakly adsorbed DTX, this precipitate was redispersed in deionized water (5.0 mL) via ultrasonication (100 kHz, 200 W/L) for 30 s and separated by centrifugation one more time. Finally, DTX@HNTs/CPS was dried using a freeze drier for 24 h.

#### Preparation of AuNPs

To a 20-mL vial containing 15.84 mL of water, 0.188 mL of 5.0 mM aqueous solution of tetracholoroauric acid was added, followed by 0.400 mL of 1 mM of freshly prepared sodium borohydride in water. The reduction to yield orange-red dispersion of ca. 10-nm AuNPs took place in several seconds. Finally, 0.188 mL of 5 mM trisodium citrate was added to stabilize the resulting AuNPs for incorporation into HNTs.

#### Preparation of DTX@HNT/Au-SORT

In a 25-mL round bottom flask, DTX@HNTs/CPS (0.050 g) was dispersed in 3.0 mL of PBS (0.1 M, pH = 8) using ultrasonication (< 15 s). To assist the dispersion process, a drop (ca. 0.05 mL) of ethanol was added into the flask during the ultrasonication. Next, the reaction mixture was stirred at 4 °C for 10 min using a salt (NaCl) ice bath. In the next step, as-prepared AuNPs (2.0 mL of the colloidal dispersion of 0.15 mM by gold) was added upon gentle stirring. Subsequently, sortilin 2D8-E3 mAb (10 μL, 50 μg/mL) was added into the reaction flask. After 2 h of gentle stirring at 4 °C in an ice bath, DTX@HNT/Au-SORT was separated via centrifugation (25,200 RCF, 5 min), the collected precipitate was washed with cold deionized water (5.0 mL), and finally freeze-dried for 48 h.

### Evaluation of DTX content in DTX@HNT/CPS

To estimate the DTX content in DTX@HNT/CPS, the latter was thoroughly ground via ball-milling (25 Hz, 2 h), and 50 mg of the ground powder was dispersed in dimethyl sulfoxide (DMSO) (5.0 mL) via ultrasonication (150 kHz, 200 W/L), at 50 °C for 1 h. The resulting mixture was vigorously stirred for an additional 1 h at the same temperature. In the next step, DTX@HNT/CPS was separated via centrifugation (11,200 RCF, 15 min) and passed through the paper filter (< 2 μm pores) to produce a clear solution. The filtered solution was then diluted with ethanol (1.0 mL to 250 mL) for UV–vis measurements, and the calibration curve constructed using absorption at 230 nm.

### Flow cytometry experiments

First, caov-4 cells (10^6^ DFU) were placed in a PBS buffer solution (0.1 M, pH = 7.4) and stained by trypan blue (1% v/v, four drops, ca. 0.2 ml total), which was diluted with Dulbecco's modified Eagle’s medium (DMEM) and a drop of Lugol's solution (ca. 0.05 mL of 5% and 10% (wt/v) aqueous solution of iodine and potassium iodide, respectively). Then, the stained cells were washed by adding 1.0 mL of PBS and gentle centrifugation (252 RCF, 5 min at 20 °C). PBS-EDTA or Accutaze enzyme was used for separation and dispersion after precipitation. Subsequently, the stained cells were counted using a fluorescence microscope: 10 μL of the cell solution was measured to contain from 1 × 10^5^ to 5 × 10^5^ cells. For blocking, 200 μL of the antibody solution (10 μg/mL) was prepared to treat the stained cells in sheep’s serum (5 wt%) at 4 °C for 30 min. The cells were then washed again two times with PBS (2.0 mL) and gently centrifuged (252 RCF) for 5 min. In the next step, the prepared cells were incubated with SORT (10 μg/mL, 200 μL) at 4 °C for 1 h, then washed two times as described above, and subjected to sheep anti-human-FITC (0.1 μg/mL) at 4 °C for 30 min. After two more washing cycles, the isotonic (0.9% w/v) saline solution was added to perform flow cytometry experiments.

### Confocal microscopy experiments

A glass test tube (13 by 100 mm, equipped with a threaded cap) was sterilized at 120 °C and used to disperse DTX@HNT/Au-SORT (0.02 mg) in DME medium (5.0 mL) via ultrasonication. Then, the DTX@HNT/Au-SORT dispersion was exposed to caov-7 cells (10^6^ DFU), at 4 °C for 1 h. A portion was withdrawn at specified time intervals and stained with crystal violet (tris(4-(dimethylamino)phenyl) methylium chloride) in DMEM (1 vol%, four drops, ca. 0.2 mL), which was diluted with a drop of Lugol's solution (ca. 0.05 mL). Finally, the stained cells were placed on the microscope glass slides and dried in a vacuum oven so that they can be used for confocal microscopy experiments.

### In vitro bioassay experiments

For in vitro studies, caov-4 and 3T3 cells (both 10^4^) were cultivated in 5.0 mL of DMEM containing 10% FBS (fetal bovine serum), under 90% humidity and 5% CO_2_ at 37 °C. Three wells of a 96-well plate were dedicated to each condition for statistical analysis following the ISO 10993:5 standard protocols; 200 μL of the tested samples (20 μg/mL) was measured into each well and incubated. To obtain well-dispersed samples in DMEM, they were subjected to 5-min ultrasonication (50 kHz) in an ice bath and sterilized via UV irradiation for 10 min. After the desired incubation time (1, 6, 24, and 72 days), the medium of each well was replaced with 100 μL DMEM containing 10% MTT (3-(4,5-dimethylthiazol-2-yl)-2,5-diphenyl tetrazolium bromide) solution, and the incubation was continued for an additional 4 h. At this point, the medium of each well was partially replaced with 150 μL of DMSO to dissolve the formed crystals. Finally, the optical density of the samples was measured by an ELISA reader at 600 nm. A dispersion of DTX@HNT/Au-SORT without cells was used as a blank control. Calculations of %cell relative viability and corresponding statistical data are reported in Additional file 1: Tables S1, S2.

## Results and discussion

### Preparation of the DTX@HNT/Au-SORT nanocomposite cargo

A key factor for the therapeutic properties of the DTX@HNT/Au-SORT composite is the ability of the cargo to efficiently penetrate the targeted cells. Given that the uniformity of HNTs is crucial for the internalization and cellular uptake, it is important to assure a good size and shape uniformity of HNTs [66]. In order to accomplish this, HNT pre-treatment was employed starting with the ball milling followed by multi-stage centrifugation and dialysis. While ultrasonication is considered to be one of the most effective methods to separate colloidal particles, the prolonged exposure is detrimental to the HNT integrity, as well as colloidal stability, so the use of ultrasonication was kept at a minimum.

For the calcination step, high-temperature treatment of HNTs is performed to enhance the interlayer volume that can be available for incorporation of target molecules, e.g. drugs [67]. For HNTs, the treatment to enhance the porosity is typically carried out in a temperature interval of 400 to 600 °C [64]. Subsequently, treatment of HNTs with acids causes activation of hydroxyl (–OH) groups (silanol and aluminol) by partial dissolution of silica to silicic acid, and thus enabling subsequent covalent attachment of target molecules [64]. In the context of our work, successful activation of hydroxyl groups is necessary for covalent bonding of the antibodies onto the HNT surface so that selective cell attachment and expression can be carried out, resulting in high levels of selected targeting. Finally, complete rinsing of HNTs is necessary since the traces of the remaining acid negatively affect the hydrogen bonding in HNTs and with HNTs due to protonation of their surface hydroxyl groups. Specifically, the reduced hydrogen bonding between the rolled layers of HNTs and DTX is detrimental for the drug loading ratio (DLR), and thus careful removal of the acid traces is important.

**Fig. 1:**
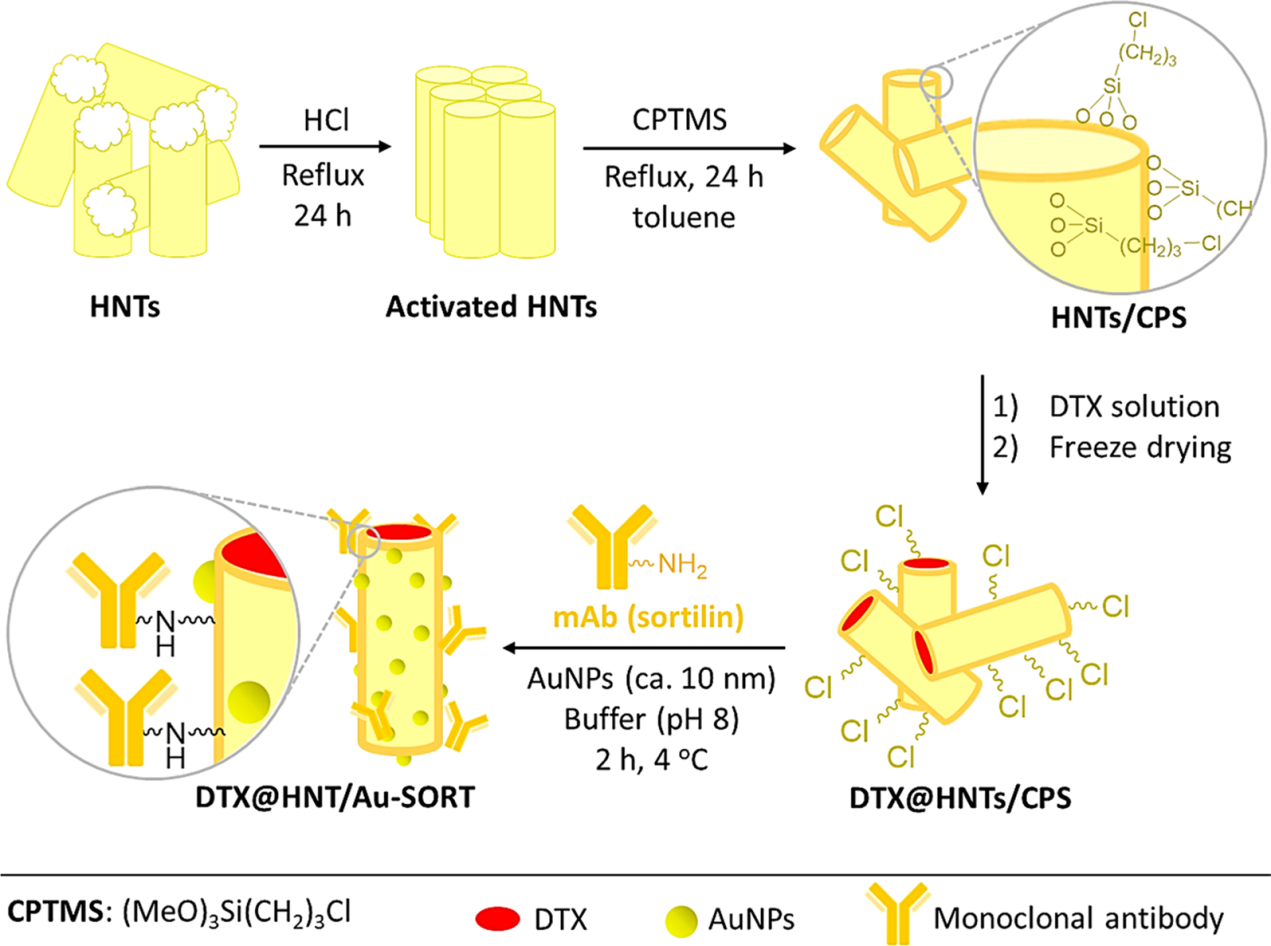
Schematic of the main steps in preparation of DTX@HNT/Au-SORT

In the first step of the DTX@HNT/Au-SORT preparation, HNTs were treated with acid and sonicated to activate the hydroxyl groups of the HNTs for subsequent reactions and to open the cavities [68]. Following the activation, HNTs were functionalized with 3-chloropropyl silane (CPS) to enable conjugation of the sortilin antibody, and to increase HNT hydrophobicity [69]. CPS provides an appropriate substrate for antibody conjugation via the covalent binding of chloropropyl groups with amine and thiol groups (antibodies with tyrosine, lysine, and cysteine) [70]. It should be noted that a partial reduction on the antibody (e.g., by dithiothreitol) is needed if conjugation from thiol sites is intended [71]. CPS increases HNT hydrophobicity by modifying the surface as has been successfully exploited previously for interactions between the internal environment of cancer cells and CPS modified HNTs [72]. The enhancement of the hydrophobicity is expected to be effective to reduce drug leaching from the designed nanoscale delivery system during circulation in the polar aqueous environment of the body [73]. After CPS modification, the HNTs are filled with DTX, conjugated with SORT and AuNPs [74], as shown in the general schematic of Fig. 1. The lysine amino groups of the antibody are then reacted with the chloropropyl groups of DTX@CPS-HNT. Figure 1 presents a general schematic of the preparation steps of DTX@HNT/Au-SORT described above.

### Characterization of DTX@HNT/Au-SORT

#### FTIR and EDX spectroscopies

**Fig. 2:**
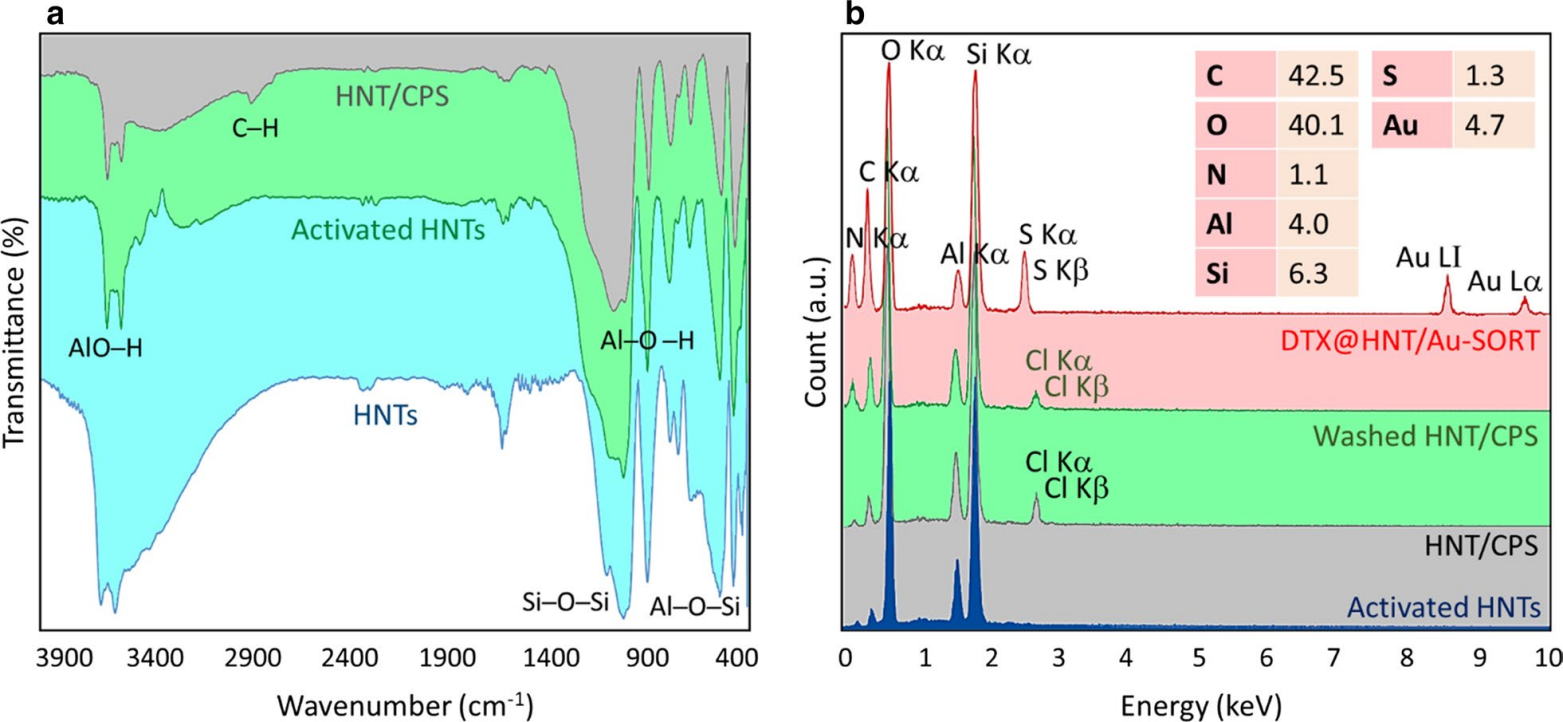
**a** Fourier-transform infrared (FTIR) spectra of non-treated HNTs, activated HNTs, and HNT/CPS; and **b** energy-dispersive X-ray (EDX) spectra of the activated HNTs, HNT/CPS before and after exhaustive washing, and DTX@HNT/Au-SORT

Halloysite activation and functionalization with CPS and SORT were monitored by Fourier-transform infrared (FTIR) and energy-dispersive X-ray (EDX) spectroscopies. As shown in Fig. 2a, two peaks corresponding to AlO–H bonds of the alumina network in the inner surface of HNTs appear at 3624 and 3695 cm^−1^ [75]. These peaks become stronger and better resolved after acid treatment in the FTIR spectrum of the activated HNTs, indicating that the HNT surfaces can interact stronger with CPS due to a larger number of polar groups [75]. Also, the peak at ca. 1600 cm^−1^ originating from the impurities in HNTs [76, 77] is significantly reduced in intensity after acid treatment. The sharp peaks at 1100, 911, and 468 cm^−1^ can be assigned to bending vibrations of Si–O–Si, Al–O–H, and Al–O–H bands, respectively [75, 78]. The surface functionalization of HNTs with CPS is confirmed by the appearance of the peak at 2939 cm^−1^, which originates from stretching vibrations of C–H bonds of sp^3^ carbon [79, 80]. The elemental analysis based on EDX data further corroborates the results of FTIR spectroscopy. As shown in Fig. 2b, the peak intensity corresponding to carbon is increased by HNT modification with CPS and SORT. Based on EDX data, 42.5% of the total weight of the HNT/Au-SORT corresponds to carbon. Coupled with the fact that antibodies remain attached after continuous washing cycles, these results support the successful functionalization of the HNTs surfaces with SORT. The appearance of sulfur signal (1.3% of the total weight) originating from cysteine also corroborates antibody attachment to HNTs. The incorporation of AuNPs has been verified by the signals at 8.7 and 9.8 keV in the EDX spectrum of DTX@HNT/Au-SORT. To confirm covalent bonding of the CPS linker onto the HNTs surfaces, a control EDX spectrum was obtained after exhaustive washing of HNTs/CPS. The amount of chlorine present in HNTs/CPS is 2.28 wt% of the total weight. The ratios of the loaded DTX and incorporated AuNPs have been estimated to be 4.7 wt% based on the EDX results, as reported in the ESI.

#### EM imaging

**Fig. 3:**
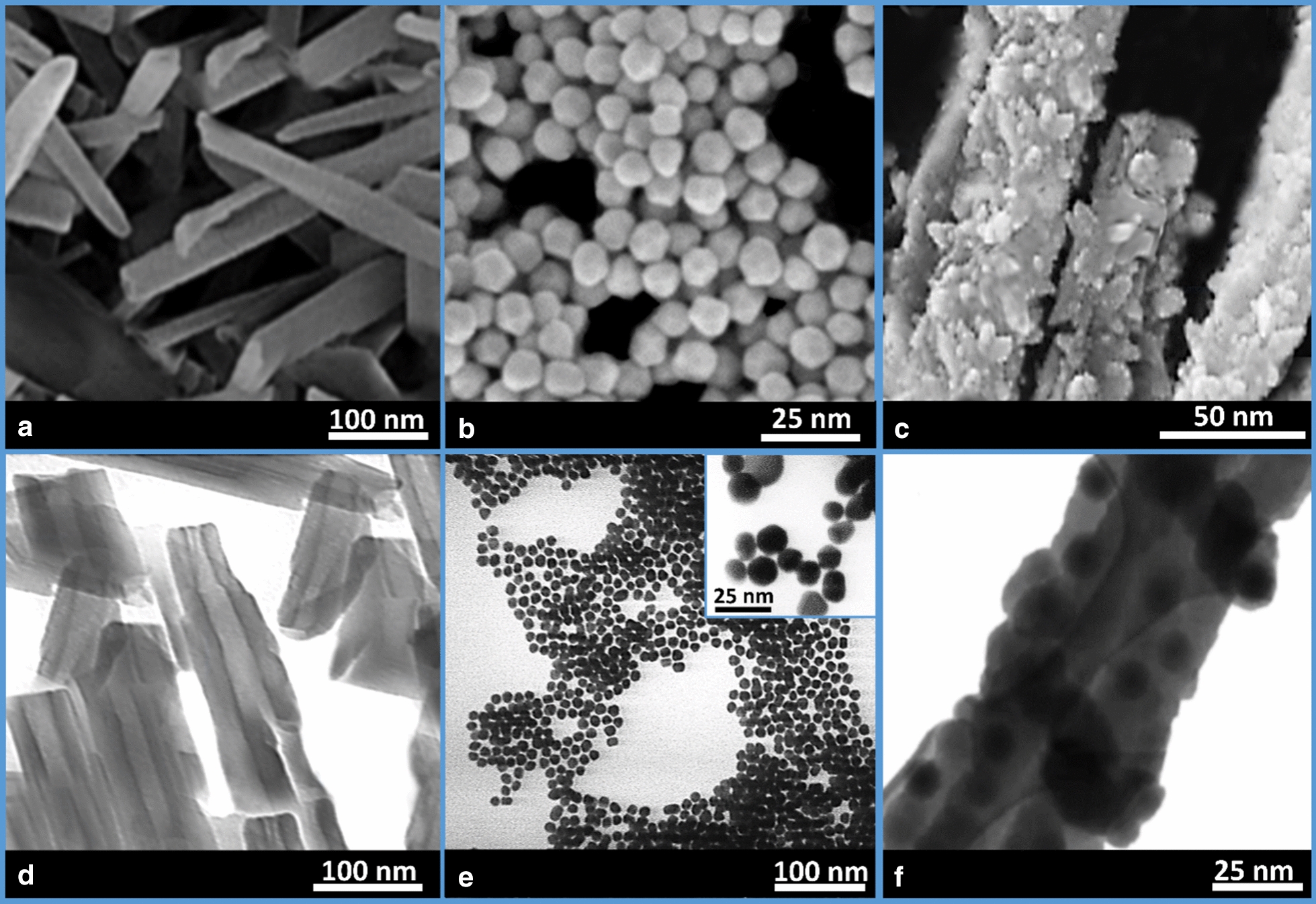
**a**–**c** Field-emission scanning electron microscopy, and **d**–**f** transmission electron microscopy images of **a**, **d** HNTs; **b**, **e** AuNPs; and **c**, **f** DTX@HNT/Au-SORT

To monitor the size and morphology of the individual components at all the synthetic stages of formation of DTX@HNT/Au-SORT, electron microscopy (EM) imaging was used. Figure 3a–c show field-emission scanning electron microscopy (FESEM) images, and Fig. 3d–f present transmission electron microscopy (TEM) images of the activated HNTs, AuNPs, and the final product, DTX@HNT/Au-SORT, respectively. From images a) and d) of Fig. 3, it can be clearly seen that HNT morphology features have openings that can be utilized for drug loading. AuNPs with a mean diameter of ca. 10 nm were used for the incorporation into the exterior walls of the HNTs. The AuNPs appear to be of a near-spherical shape, but at higher resolution in the image of Fig. 3b, the facets of the polyhedral AuNPs originating from the fcc structure of gold are apparent. AuNPs were prepared using a synthetic procedure similar to those used for small seeds via reduction with borohydride with no inherent shape selection. All possible seed geometries were present (cubooctahedral, icosahedral and some decahedral) based on our studies of similar AuNPs. Importantly, citrate was introduced to enhance the colloidal stabilization essential for the formation of composites in high yield. Figure 3b, e also confirm the average diameter of AuNPs of ca. 10 nm and their near-spherical shape. Figure 3c shows that the roughness of the outer surfaces of HNTs is significantly increased after the incorporation of AuNPs. According to the literature reports, the exterior surface of the HNTs is described as mesoporous [70], so AuNPs can be expected to incorporate into these pores. Indeed, the TEM image of Fig. 3f clearly demonstrates the incorporation AuNPs into the pores on the outer surfaces of HNTs, where the darker spots in DTX@HNT/Au-SORT correspond to AuNPs due to gold’s high atomic number. To further characterize the HNT porosity, nitrogen adsorption/desorption experiments were performed with Brunauer–Emmett–Teller (BET) surface area analysis of HNT samples after mechanical and chemical treatments. As shown in Additional file 1: Figure S1, type IV isotherm characteristic of mesoporous materials is observed for HNTs, with the pores varying in the range from 8 to 12 nm and the average pore diameter evaluated to be ca. 9.2 nm (the data accompanied Additional file 1: Figure S1). These pore size values are comparable with the mean diameter of the AuNPs (ca. 10 nm based on DLS data), so considering that the fraction of the pores occupied by AuNPs is low, the larger pores should accommodate AuNPs well, as it was observed experimentally.

#### Zeta sizer and water contact angle data

**Fig. 4:**
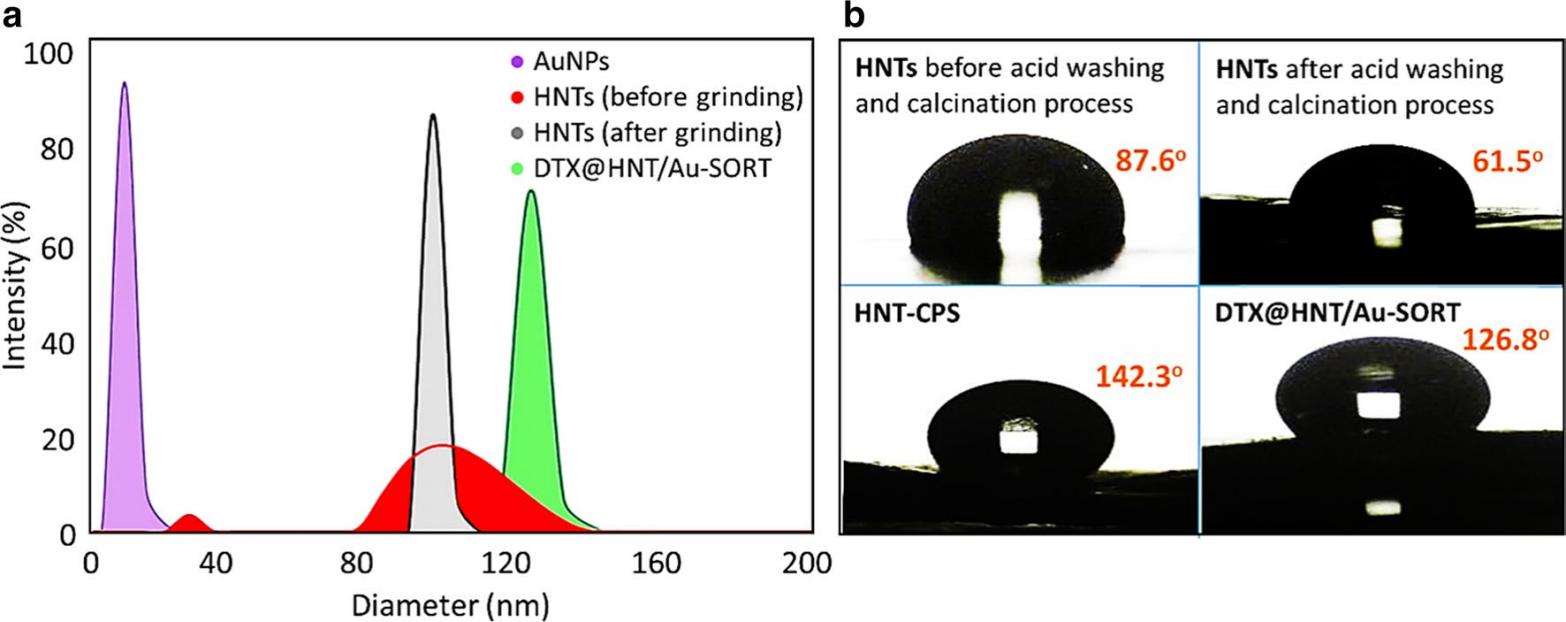
**a** Size distribution plots for AuNPs, HNTs and DTX@HNT/Au-SORT derived from the DLS data; and **b** water contact angle data for HNTs at different stages of modification

The stability and average size of particles in solution through all synthetic steps of DTX@HNT/Au-SORT formation were monitored using a zeta-sizer. Zeta-sizer data provide more detailed information on the average size of the nanostructures in their dispersed state. These data nicely complement the information from EM imaging, where samples were dried in a vacuum so that potential drying artifacts can be identified and excluded. Based on dynamic light scattering (DLS) data (Fig. 4a), the mean size of the synthesized AuNPs is ca. 10 nm. The polydispersity index (PDI) for the synthesized AuNPs is measured to be 1.2, confirming their reasonable size uniformity and colloidal stability in dispersions. What is also clearly demonstrated by the DLS measurements is that HNTs have higher PDI before grinding and calcination. After HNT grinding via ball-milling, significantly higher uniformity is attained, with an average HNT size of ca. 110 nm based on DLS data calculated for the equivalent hydrodynamic diameters based on the measured diffusion coefficients. The average size of the HNTs and the peak PDI increase slightly after the incorporation of AuNPs into the HNT pores, while corroborating the stability and dispersibility of the resulting composites. Finally, the mean size of the prepared DTX@HNT/Au-SORT particles can be estimated to be ca. 130 nm, which is a near-optimal size for in vivo transport and cellular uptake [81].

According to the reported data [82, 83], the hydrophobic properties of the cargo are considered to be one of the most important factors for drug delivery to the cancer cells due to their lipophilic environment. Furthermore, since the cargo is circulated in the aqueous environment of the body, drug leaching is strongly inhibited by the hydrophobic surfaces of drug carriers [84]. In this context, we utilized CPTMS for the surface modification of HNTs to increase hydrophobicity. Immediately, it was observed that the CPS-modified HNTs could hardly be dispersed in water. These observations corroborate that the hydrophobicity of the CPS-modified HNTs is enhanced. To quantify these observations, measurements of water contact angles were performed to compare HNTs before and after modification with CPTMS. Figure 4b shows that the contact angle is reduced from 87.6° to 61.5° by acid washing and calcination. It is reasonable to consider that the hydrophilicity of the HNTs surfaces is enhanced through activation of the hydroxyl groups of the silica network in the exterior surface. The contact angle values increased significantly to 142.3° after HNT surface modification with CPS that attests the effectiveness of this procedure. Lastly, the contact angle is decreased to 126.8° through surface functionalization with the SORT antibodies that are more polar. Overall, these results confirm that DTX@HNT/Au-SORT exhibits significantly less hydrophilic interactions, so it can interact less with blood serum and have minimized DTX leaching.

### Therapeutic properties of DTX@HNT/Au-SORT

#### Drug release by plasmonic heating (PPTT) of AuNPs

**Fig. 5:**
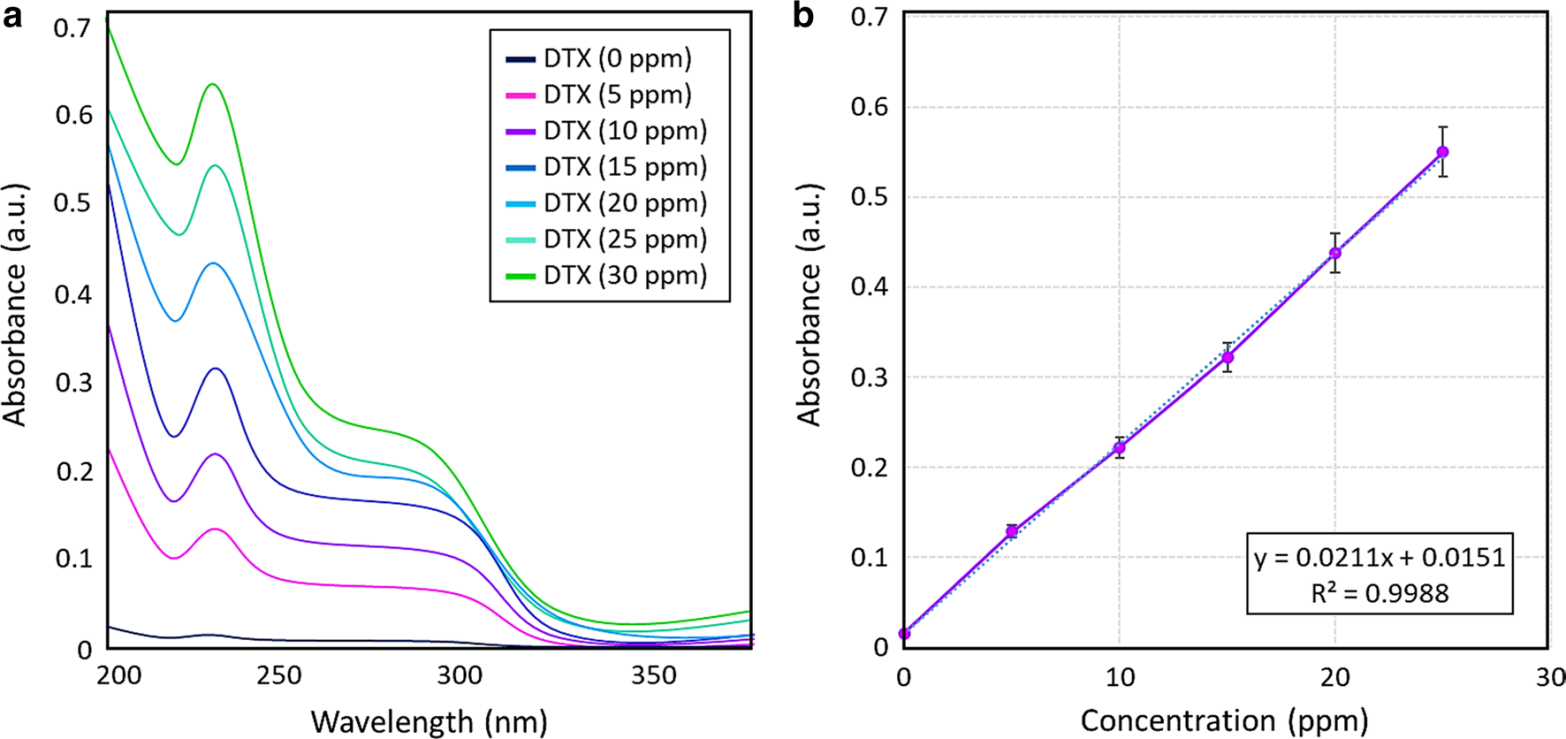
**a** UV–vis spectra, and **b** calibration curve for the determination of DTX concentration constructed using standard DTX samples in ethanol (bars show relative errors of triplicate measurements)

DTX release process from DTX@HNT/Au-SORT was carefully monitored in simulated blood serum and the internal environment of cancer cells to understand and to characterize the behavior of the developed nanocomposite drug carrier. At neutral pH, the cargo is expected to circulate for a relatively long time in the blood serum [85]. After the internalization of the cargo, the pH of the internal environment of the cancer cells is reduced to acidic values due to the secretion of lysosomal enzymes [86]. Therefore, the effect of pH on the cargo stability and drug release process needs to be studied. The DTX release process from HNTs decorated with AuNPs was also investigated at temperatures higher than 37 °C (typical body temperature). For this purpose, the irradiation of AuNPs by white LED light was used. To properly account for the amount of DTX loaded into HNTs, a previously reported procedure was employed [87]. Briefly, a calibration curve was produced based on six standard samples with DTX concentrations of 5, 10, 15, 20, 25, and 30 ppm (Fig. 5a). From the resulting linear calibration curve with the R-value of 0.9988 (Fig. 5b), the concentration of DTX in the test samples (C) could be determined using the following Eq. (1):

1 $$C \left( {ppm} \right) = \frac{{A \left( {a. u.} \right) - 0.0151}}{0.0211}$$

#### Evaluation of the DTX content in DTX@HNT/Au-SORT

The exact amount of the DTX incorporated in DTX@HNT/Au-SORT carriers was found to be slightly lower than the released amount because of the DTX entrapment in the HNT interlayer space caused by DTX binding to silanol groups [88]. At the same time, DTX can be completely released when an appropriate solvent (e.g., DMSO) is used. To estimate the DTX amount as precisely as possible, 50 mg of DTX@HNT/Au-SORT was thoroughly ground and dispersed in 5.0 mL of DMSO via ultrasonication, and stirred for 1 h at 37 °C. Finally, the solid fraction was separated by filtration and the resulting supernatant was diluted 250 times with PBS (pH = 6.8, 0.1 M), and then studied by UV–vis spectroscopy. Assuming that most of the DTX@HNT/Au-SORT is DTX, dilution to 40 ppm is reasonable for UV–vis characterization. A DTX-free sample (HNT/Au-SORT, 50 mg in 5.0 mL) was also studied along with the DTX@HNT/Au-SORT sample, as a control. In fact, the background of the UV–vis absorbance spectra has been obtained from HNT/Au-SORT measured at the same conditions to minimize the effect of SORT leaching and scattering from the particles. Based on the calibration curve at 230 nm (Fig. 7b), the amount of the DTX loaded in DTX@HNT/Au-SORT can be estimated to be (28.0 ± 1.2) wt%, as shown below:

$$Concentration\;of\;the\;sample = \frac{{50\;mg \left( {particles} \right)}}{{5.0\;mL \left( {DMSO} \right)}} = 10\;\frac{mg}{{mL}} = 10{,}000\;ppm$$

$$Considering\;that\;all\;10{,}000\;ppm\; is\;DTX\mathop{\longrightarrow}\limits^{{diluted \left( {1.0\;mL to 250\;mL} \right)}} 40.0\;ppm$$

$$A \left(a. u.\right)=0.251 \stackrel{from eq. 1}{\Rightarrow } C\left(ppm\right)=11.2$$

$$\% \;Estimated\;DTX\;content = \frac{11.2}{{40.0}} \times 100 = 28.0\%$$

**Fig. 6:**
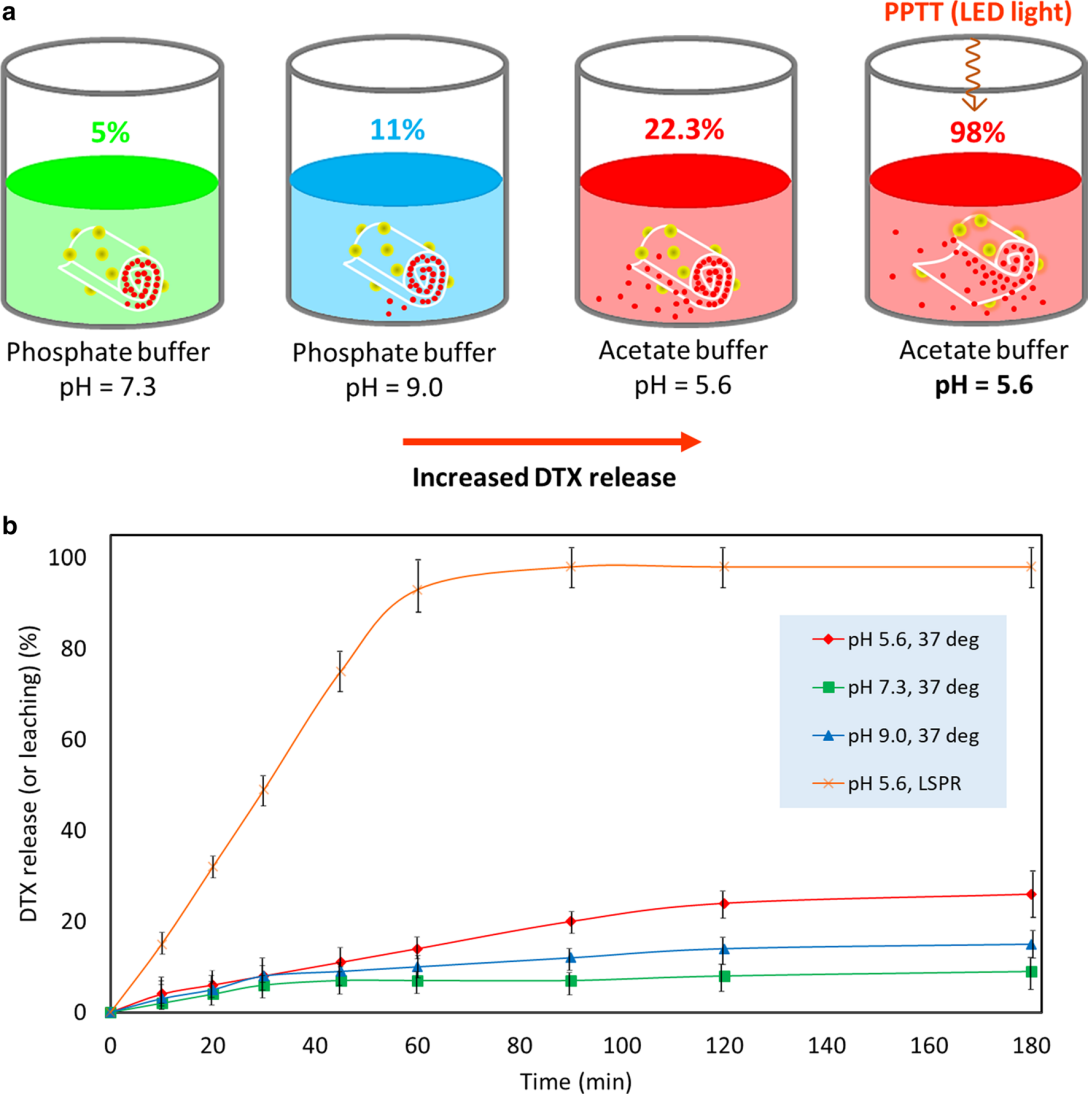
**a** Schematic of different experimental conditions studied for the drug release process from DTX@HNT/Au-SORT, and **b** DTX release profile from DTX@HNT/Au-SORT in conditions described in **a**, see Table 1 for more information

Figure 6a presents a schematic of the drug release screening. Four samples with the same amount of DTX@HNT/Au-SORT (50 mg per 5.0 mL) were prepared in different buffer media at different pH values. The released DTX values were estimated relative to the calculated DTX content in DTX@HNT/Au-SORT (28 wt% or 11.2 ppm). As shown in Fig. 6a and also presented in Table 3 (entry **2**), the highest DTX release value of (98 ± 1.2)% is observed in acidic conditions under irradiation with white LED light (7 W). The leaching of AuNPs from the cargo was also studied by inductively coupled plasma mass spectrometry (ICP-MS) analysis. No significant release of AuNPs (1.4 ppm) was found after 180-min stirring. At the same time, as reported in Table 3, (22.3 ± 0.6)% DTX release occurs in the acidic environment in the absence of light irradiation (entry **1** in Table 3) that can then serve as an independent factor to trigger DTX release. It was also observed that more DTX is released at basic pH (11.0 ± 1.5)% compared to neutral conditions (Table 3, entry **4**). The UV–vis spectra characterizing the drug release study are shown in Additional file 1: Figure S2.

**Table 3:** DTX release data from DTX@HNT/Au-SORT in different experimental conditions in vitro

Entry	Conditions	Released (or leached) DTX (%)	Time (min)	Relative error (%)
1	AcBS (pH 5.6), 37 °C, no light	22.3	120	3.0
2	AcBS (pH 5.6), LED irradiation	98.0	90	1.2
3	PBS (pH 7.3), 37 °C, no light	5.0	120	30.0
4	PBS (pH 9.0), 37 °C, no light	11.0	120	14.0

*AcBS* acetate-buffered saline, *PBS* phosphate-buffered saline (both 0.1 M)

#### Effects of PPTT heating on physical expansion of HNTs

**Fig. 7:**
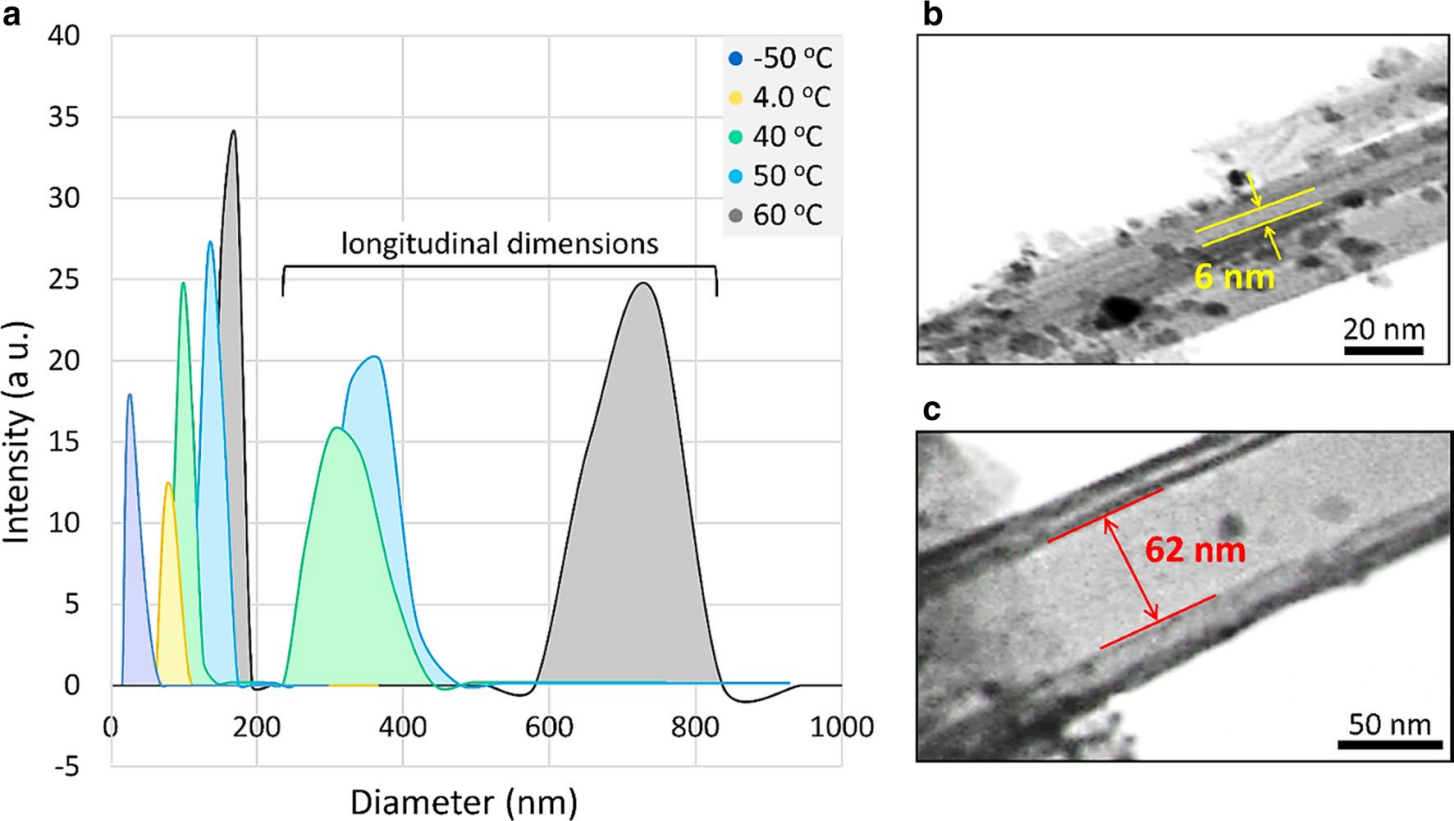
**a** DTX@HNT/Au-SORT size distribution at different experimental conditions based on DLS data, and **b**, **c** TEM images of DTX@HNT/Au-SORT at **b** low temperature of ca. − 50 °C and **c** high temperature of ca. 50 °C

**Fig. 8:**
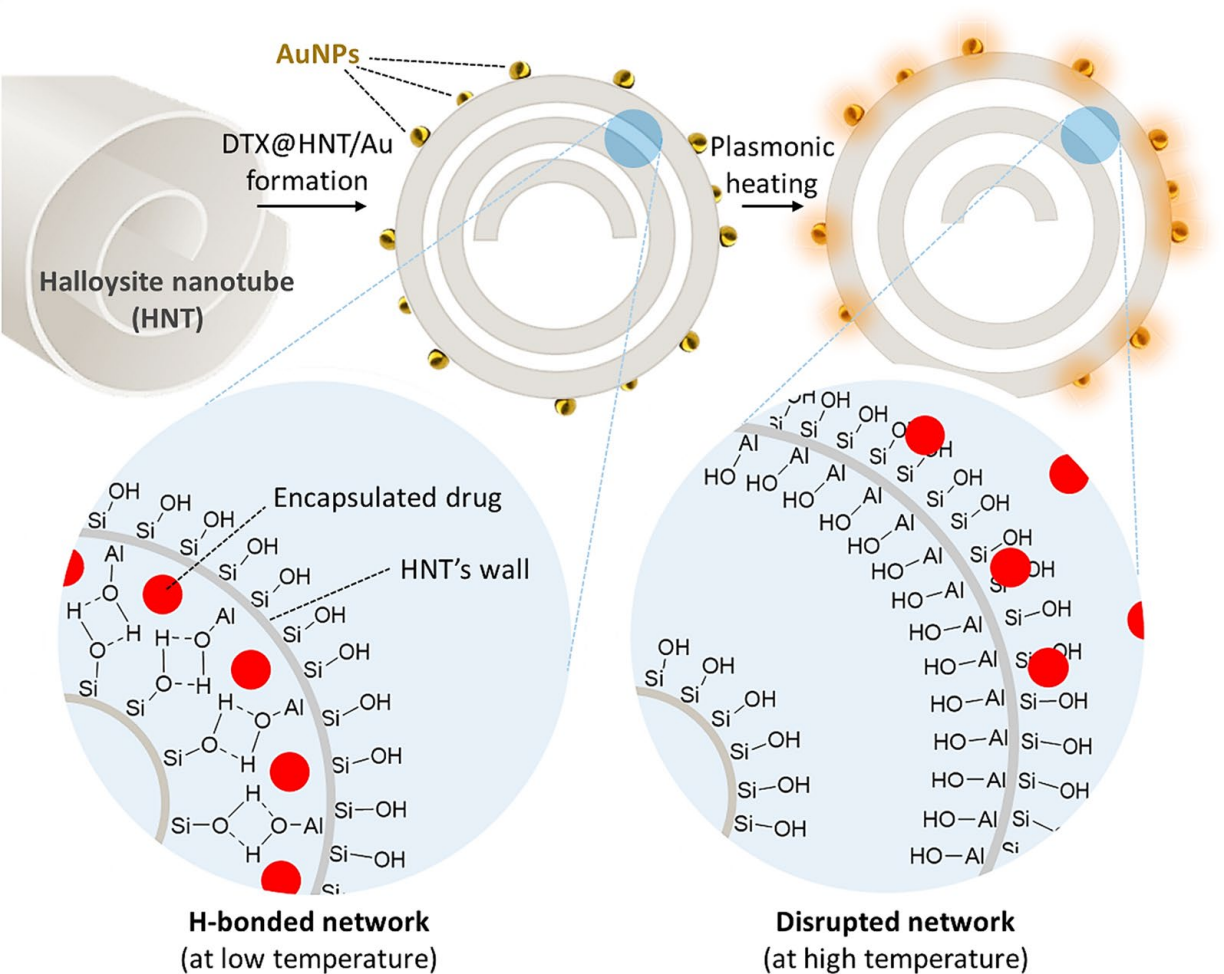
Schematic structure of HNTs rolled tubular morphology decorated with AuNPs in the outer layers (top): hydrogen-bonded network with drug encapsulation at low temperatures (bottom left), and release at high temperatures (bottom right)

To experimentally investigate the physical expansion of the HNTs upon heating, which is used as a primary trigger of the controlled DTX release from the DTX@HNT/Au-SORT system, the changes of the HNT hydrodynamic size were monitored by DLS. For this purpose, a series of dilute dispersions (ca. 0.5 mg/mL) were prepared and measured at different temperatures. As reported in Table 4, a wide thermal range from − 50 to 60 °C was explored to clearly demonstrate the temperature-driven changes of the particle size of HNTs. The lowest average size of ca. 55 nm was observed at the lowest temperature (− 50 °C) in ethanol, implying that the DTX-wrapped in HNTs layers will likely be expelled through the contraction of the walls at low temperatures. The values for the average HNT size gradually increased at higher temperatures, reaching ca. 151 nm at 50 °C and ca. 171 nm at 60 °C. The observed increase in the HNT size originates from the expansion of the rolled multi-walled HNTs. Importantly in the context of this work, the heating can be attained by virtue of the PPTT effect through irradiation of AuNPs incorporated in HNT pores of DTX@HNT/Au-SORT. Based on the data shown in Table 4 (entries 4–6), ca. 20 nm expansion can be achieved after LED irradiation for 5 min due to the light absorption through the LSPR of AuNPs. Figure 7a shows the DLS-based size distribution curves of the HNT colloidal dispersions at different temperatures. It can be seen in this figure that the sharp peaks with the large PDI values above 200 nm are observed that can be potentially attributed to the HNT longitudinal parameters corresponding to the slower diffusion values measured by DLS, with the understanding of limitations in that the values are estimated by the DLS software using equivalent hydrodynamic spheres. The expansion of the interior space of the HNTs at different temperatures was also investigated by TEM imaging. As illustrated in Fig. 7b, c, the diameter of the interior tubular space (the distance between two HNT layers in the internal spaces) is ca. 6 nm at lower temperatures (− 50 °C), whereas the layers are separated to ca. 62 nm at 50 °C upon PPTT heating, as shown in Fig. 7b, c, and illustrated schematically in Fig. 8.

**Table 4:** Physical expansion of the multi-walled HNTs in different conditions characterized by DLS

Entry	Average particle size (nm)	Temperature of the colloidal solution (°C)	Experimental conditions^a^
1	55	− 50	Dry ice/acetone bath, ethanol
2	72	4	Ice/salt bath, ethanol
3	110	25	Room temperature in water^b^
4	134	40	White LED irradiation for 5 min^c^ in water
5	151	50	White LED irradiation for 10 min in water
6	171	60	White LED irradiation for 15 min in water

^a^Dispersions were subjected to sonication for 5 min prior to Dynamic Light Scattering (DLS) measurements

^b^The DLS size distribution data for this condition are shown in Fig. 6a

^c^Heating through the PPTT effect of AuNPs incorporated into HNTs

#### Role of acidic pH and PPTT synergy for controlled DTX release

**Fig. 9:**
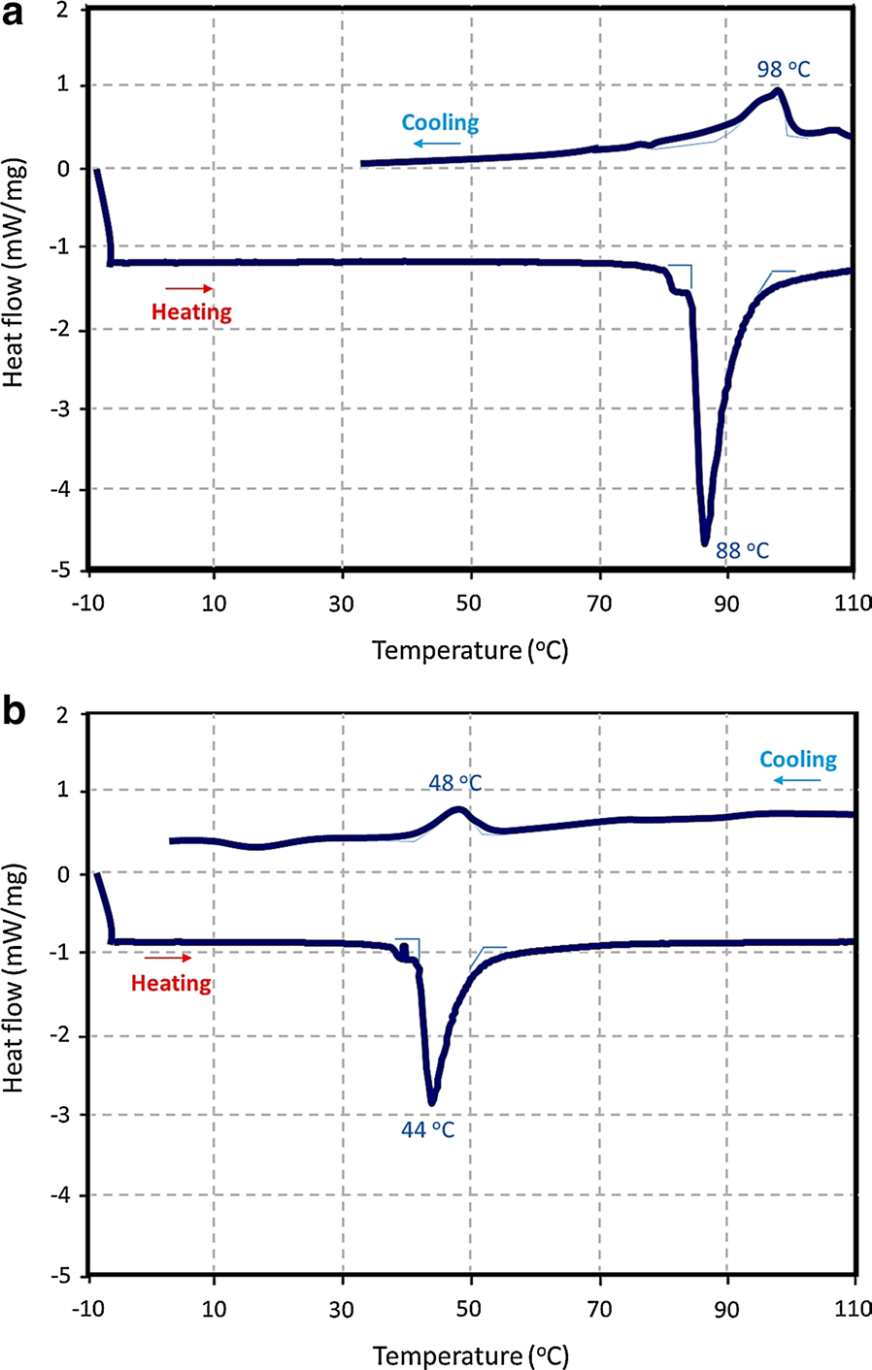
Differential scanning calorimetry (DSC) data for the HNT dispersion in a mixture of ethylene glycol with buffers (2:1 by volume): **a** in neutral environment (PBS, 0.1 M, pH = 7.3) and **b** in acidic environment (AcBS, 0.1 M, pH = 5.6)

To investigate the synergy of the PPTT heating and acidic environment for the DTX release, HNT dispersions were studied using differential scanning calorimetry (DSC) [89]. In addition, the thermal stability of HNTs was confirmed using thermogravimetric analysis, TGA, (Additional file 1: Figure S3). The DSC data presented in Fig. 9 clearly demonstrate the significant effect of the acidic pH on the breaking of hydrogen bonds that held together HNT layers. For HNT dispersions in neutral pH (PBS buffer, 0.1 M, pH = 7.3), the endothermic peak in Fig. 9a has the onset at ca. 86 °C with the well-defined maximum observed at ca. 88 °C. At the same time, an analogous transition in the acidic environment (AcBS, 0.1 M, pH = 5.6) is observed with the peak at appreciably lower temperatures (ca. 44 °C) and with the smaller magnitude (ca. 30% less in enthalpy), as shown in Fig. 9b. HNT hydrogen bonds at lower pH are significantly disrupted by oxygen protonation and thus are breaking (melting, if to make an analogy with DNA) at significantly lower temperatures.

The observed higher temperature of the restoration of hydrogen bonding relative to the melting is likely due to partial rebuilding of HNT layers in more facile pathways, as corroborated by the correspondingly smaller values of the peaks. An additional factor is ethylene glycol, added to perform DSC experiments, which can assist the HNT layer rebuilding at higher temperatures (and energies) due to its multiple hydrogen bonding. Ethylene glycol (2:1 volume ratio to buffers) has been used to minimize solvent evaporation.

Importantly for the practical realization of the DTX@HNT/Au-SORT synergy, low temperatures of the disruption of the HNT hydrogen bonding in DTX@HNT/Au-SORT at low pH can be conveniently reached through the PPTT-heating of the AuNPs [6]. This enables facile implementation of the DTX@HNT/Au-SORT design based on the synergy of the PPTT and targeted release of DTX incorporated in HNTs.

#### Evaluation of SORT conjugation to HNTs

**Fig. 10:**
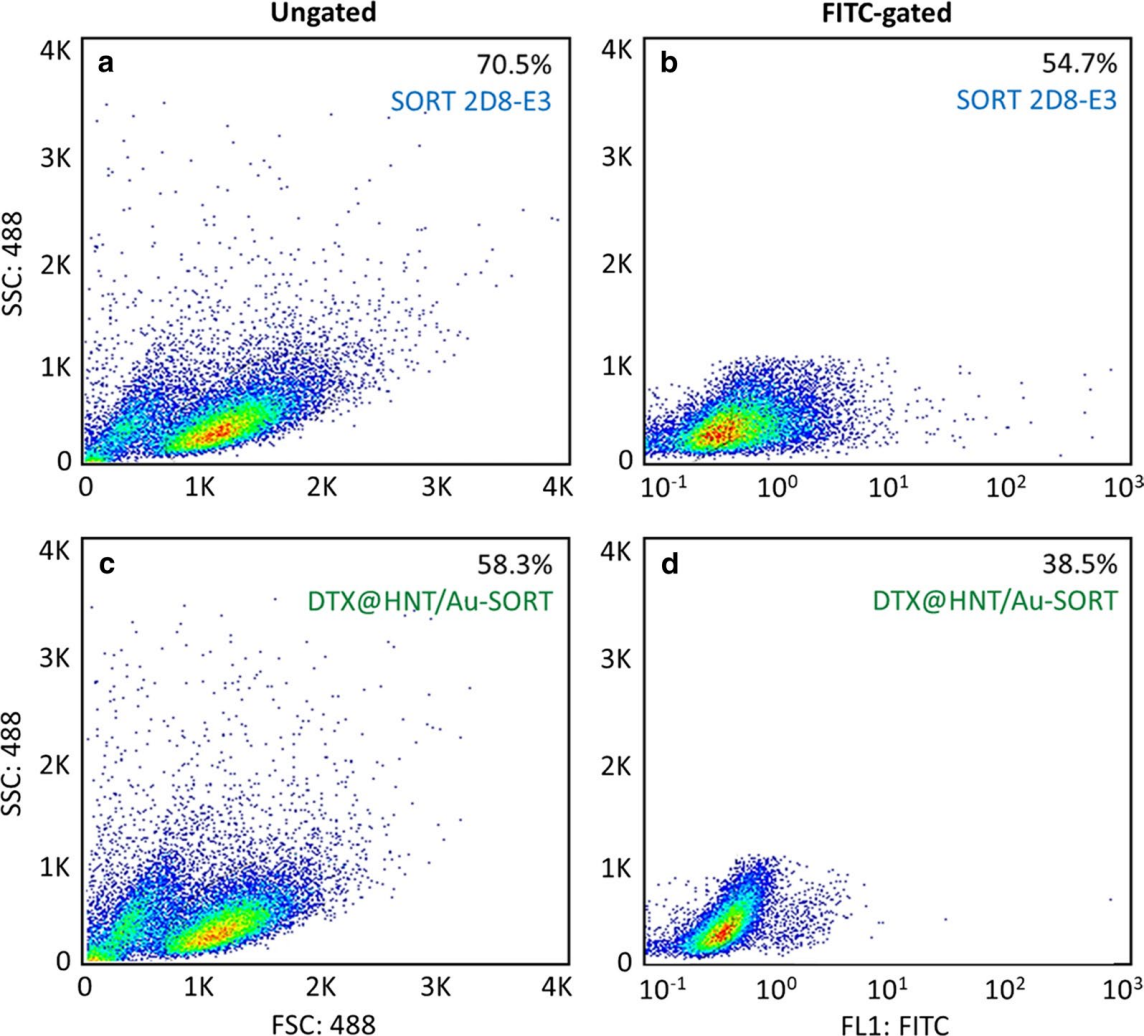
Flow cytometry FSC × SSC density plot of **a**, **c** ungated subsets with 488 nm excitation, and **b**, **d** FITC-gated caov-4 (ATCC HTB76) ovarian cancer cells in the presence of: **a**, **b** sortilin 2D8-E3 mAb as the control, and **c**, **d** DTX@HNT/Au-SORT

Flow cytometry gating was used to determine the cell expression ratio in the presence of DTX@HNT/Au-SORT compared with the individual sortilin 2D8-E3 antibody on the caov-4 cell line [90]. From the data of FSC × SSC density plot (488 nm subset) after fluorescein isothiocyanate (FITC) gating, the total expression of (77.6 ± 1.6) % by caov-4 cells for sortilin 2D8-E3 was found (Fig. 10a, b). As expected, there was a drop of ca. 11% in the antibody expression value for the DTX@HNT/Au-SORT because SORT becomes less available when coupled with HNTs. At the same time, the results of flow cytometry confirmed that (66 ± 2.1)% cell attachment has been observed after conjugation of SORT to HNTs (Fig. 10c, d). The percentage of the expressed antibodies was calculated using Eq. 2. A detailed description of the flow cytometry experiments is provided in “Experimental” section.

2 $$\% {\text{Cell attachment}} = \frac{{percentage\,of\,the\,living\,cells\,after FITC{ - }gating}}{percentage\,of\,the\,living\,cells\,before\,gating} \times 100$$

#### Internalization and cell uptake of DTX@HNT/Au-SORT

**Fig. 11:**
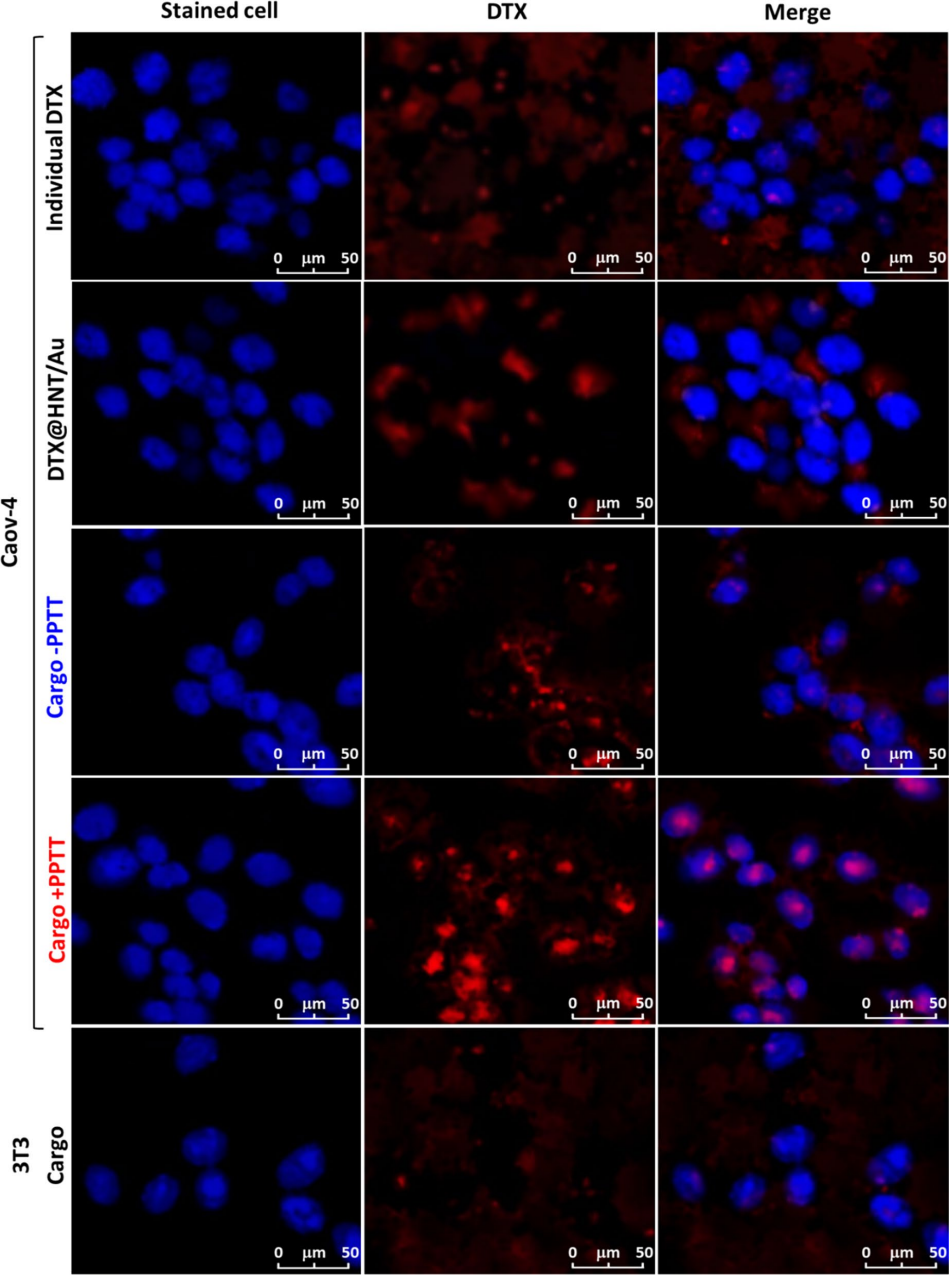
Confocal microscopy images of the caov-4 (cancerous) and 3T3 (normal) cells stained by crystal violet, in the presence of the individual DTX and DTX@HNT/Au-SORT cargo, after 90-min incubation to 7-W white LEDs to demonstrate the effect of the PPTT-release conditions

In order to perform a precise assessment of the cellular uptake, confocal microscopy was employed. As illustrated in Fig. 11, the stained caov-4 cells (blue) and DTX@HNT/Au-SORT cargo (red) can be successfully distinguished with high contrast. Both studied cell lines (caov-4/ATCC HTB76 and 3T3) were stained by crystal violet color (λ_max_ = 592 nm), and the cargo particles were detected by DTX (λ_max_ = 228 nm). The confocal images of Fig. 11 show that there is no selective cell adhesion of the neat DTX and DTX@HNT/Au to the caov-4 cells. Whereas it is clearly observed that the cargo particles (mapped in red) have been aggregated around the cells. These observations confirm that after a 45-min incubation at 37 °C the DTX@HNT/Au-SORT is well co-localized with the caov-4 cells through the adhesion onto the receptors at the surface of the cells. Furthermore, the efficiency of the PPTT-triggered drug release was evaluated in the conditions of light irradiation (white LED, 7W) treated with the DTX@HNT/Au-SORT. As can be seen in the merged images, a significant release of DTX has been occurred inside the cells under PPTT condition (cargo + PPTT). The significant value of the released DTX is discerned with the intense red color in the image of the DTX and the pink color in the merge image. This amount is very low in the absence of the PPTT conditions (cargo −PPTT). In fact, the confocal images have revealed that there would be substantial states of both cell internalization through the expression of the SORT antibodies onto the surfaces and also the controlled drug release in the PPTT conditions through photothermal heating of the AuNPs. The effect of the DTX@HNT/Au-SORT cargo on the normal cells (3T3) was also investigated by the confocal imaging method. As it can be observed in the last row (3T3 cargo) of Fig. 11, no noticeable cell adhesion and further internalization occurred on the 3T3 cells. These findings highlight the effectiveness of the bio-conjugation of the SORT antibodies in the demonstrated targeted therapy.

#### Evaluation of DTX@HNT/Au-SORT efficiency, selectivity, and cytotoxicity as a therapeutic agent

**Fig. 12:**
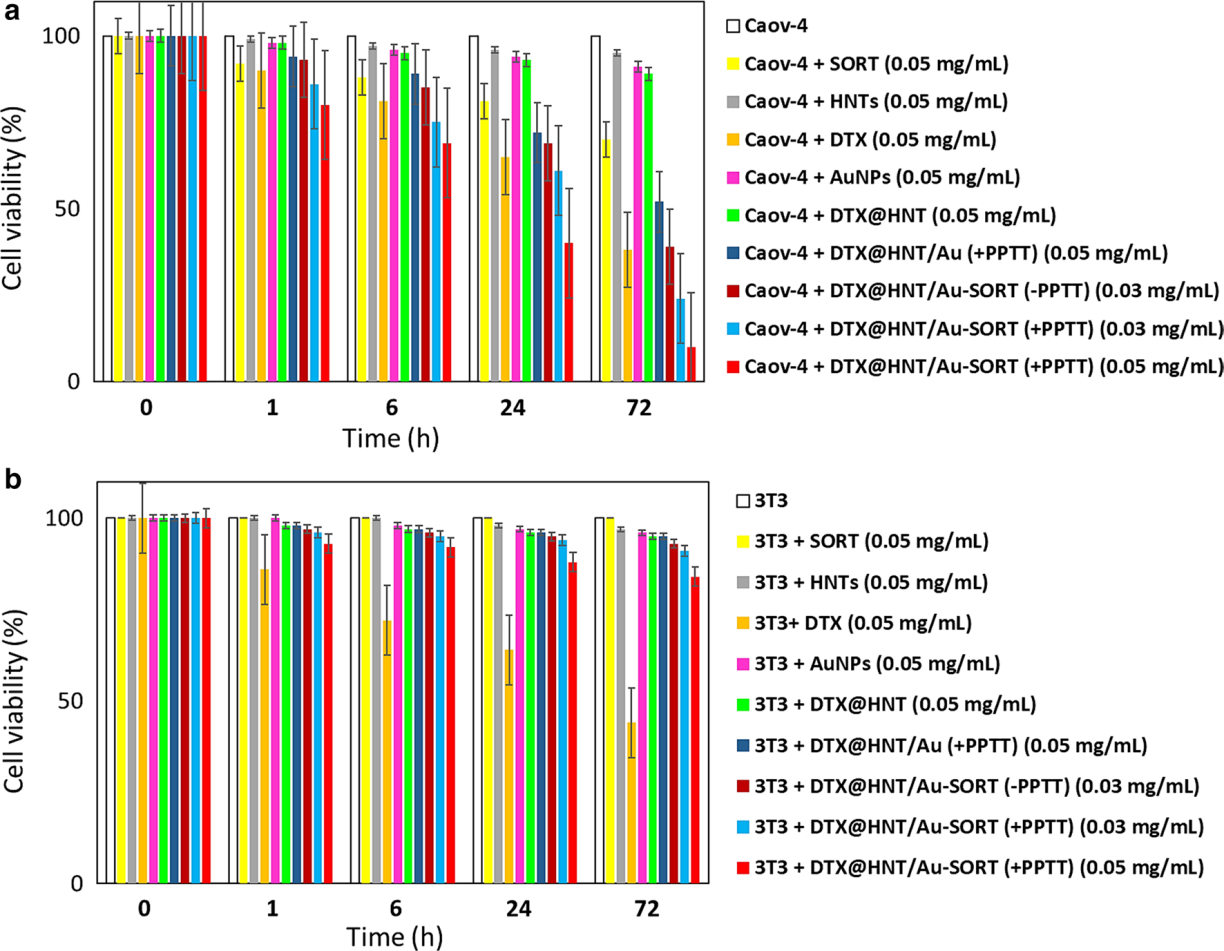
Comparative cytotoxicity data obtained in the MTT tests with **a** caov-4 ovarian cancer cell line, and **b** 3T3 normal human cell line (fibroblasts) after 72 h of exposure to DTX@HNT/Au-SORT at two different concentrations of 30 and 50 μg/mL, as well as pure SORT mAb, HNTs, DTX, and AuNPs, and DTX@HNT and DTX@HNT/Au composites. PPTT conditions were achieved using white LED (7 W) for 10 min every two hours. Error bars show %relative errors reported for triplicate measurements (see Additional file 1: Tables S1, S2)

To perform a quantitative evaluation of cytotoxicity, cell killing potency (CKP), and selectivity of DTX@HNT/Au-SORT, methyl tetrazolium (MTT) assay tests were performed using two cell lines: caov-4 cancerous cells and 3T3 normal human cells. As shown in Fig. 12a, caov-4 cells were treated with two different concentrations of DTX@HNT/Au-SORT (30 and 50 μg/mL), with a total exposure time of 72 h. Comparison experiments included neat cultivated caov-4 ovarian cancerous cells and SORT mAb, as well as other individual components: HNTs, DTX, and AuNPs, and binary nanocomposites: DTX@HNT and DTX@HNT/Au with the same concentration (50 μg/mL) to serve as essential controls. Figure 12 shows that both caov-4 and 3T3 cells have similar CKP values upon exposure to neat DTX for 72 h: (62 ± 0.5)% and (56 ± 0.4%), respectively. These high values of CKP for non-cancerous cells highlight the negative side effects of the administration of neat DTX [91]. In contrast, it was clearly observed that DTX@HNT/Au-SORT has selectively affected only the caov-4 cells for all concentrations used in this study. Figure 12a demonstrates that the viability values for caov-4 cells upon treatment with DTX@HNT/Au-SORT (50 μg/mL) are only (10 ± 0.3)%, under PPTT-release conditions (labeled as “+PPTT”) using white LED irradiation (7 W). At the administered dosage of 30 μg/mL of DTX@HNT/Au-SORT, the viability values are (39 ± 0.5)% in the absence of the white LED light (“−PPTT”) and (24 ± 0.6)% under PPTT conditions. Whereas (84 ± 4.1)% viability was observed for 3T3 cells upon treatment with DTX@HNT/Au-SORT at 50 μg/mL for the same exposure time (72 h) and PPTT-release condition (Fig. 12b). From the comparison of the therapeutic effect of DTX@HNT/Au and DTX@HNT/Au-SORT on caov-4 cells, a significant role of SORT mAb can be highlighted: CKP values of (48 ± 0.4)% and (90 ± 0.3)% were observed for the same dosage of DTX@HNT/Au and DTX@HNT/Au-SORT, respectively. Thus, the observed difference can be attributed to the role played by SORT mAb. The data of Fig. 12 demonstrate that DTX is selectively delivered to caov-4 tumor cells using the developed DTX@HNT/Au-SORT nanomedicine and PPTT-release strategy, with a comparatively negligible effect on the normal human cells observed. This minor effect likely originates from DTX leached from DTX@HNT/Au-SORT. We have also confirmed that individual components of DTX@HNT/Au-SORT, e.g., AuNPs, exert only a minor effect on the cell viability. At the same time, the selective delivery of DTX results in low negative side effects compared to the treatment with neat DTX.

## Conclusion

Synthetic design and application of the nanotherapeutic, DTX@HNT/Au-SORT, for selective targeting of ovarian tumor cells have been successfully demonstrated. DTX@HNT/Au-SORT is designed to deliver an excellent functional synergy of all its constituents: HNTs with their tunable interlayer interactions serving as a container for DXT that is capable of the triggered release; AuNPs enabling targeted release through plasmonic heating (PPTT); and SORT being responsible for the targeted specificity to ovarian tumor cells. DTX@HNT/Au-SORT features high thermal stability due to its inorganic scaffold and low leaching of DXT in physiological conditions attained by HNT hydrophobization with CPS. The role of the physical expansion of HNT layers and disruption of HNT interlayer bonding in the controlled DTX release have been elucidated, with the strategic combination of the acidic pH and PPTT effect resulting in lowering the transition by more than 40 °C from 88 to 44 °C that can be comfortably reached by the PPTT heating. Consequently, a therapeutic synergy of the targeted release could be demonstrated by the PPTT action of AuNPs under light irradiation, with the plasmonic heating assisting DTX unloading from DTX@HNT/Au-SORT. The selective therapeutic action of DTX@HNT/Au-SORT is confirmed with caov-4 ovarian cancer cells, achieving ca. 10% cell viability at 50 μg/mL. Low cytotoxicity of DTX@ HNT/Au-SORT is asserted with ca. 84% viability of 3T3 normal human cell line for the same dosage of DTX@ HNT/Au-SORT. Overall, the designed DTX@HNT/Au-SORT is promising for further development as a targeted nanotherapeutic for ovarian cancer.

## Supplementary Information

**Additional file 1: Figure S1.** BET analysis: (a) N_2_ adsorption/desorption, and (b) pore diameter of HNTs. **Figure S2.** UV–vis spectra for quantification of in vitro DTX release from DTX@HNT/Au-SORT at different experimental conditions. **Figure S3.** TGA curve of HNTs performed under argon atmosphere. Calculations of cell viability for ables S1 and S2 using the data of Figure S1. **Table S1.** Optical density data of the wells containing caov-4 cells for in vitro bioassay experiments. **Table S2.** Optical density data of the wells contating 3T3 cells for in vitro bioassay experiments. Calculation of the ratios of different components in DTX@HNT/Au-SORT based on EDX analysis.

## Data Availability

Not applicable.

## References

[1] Krukiewicz K, Zak JK (2016). Biomaterial-based regional chemotherapy: local anticancer drug delivery to enhance chemotherapy and minimize its side-effects. Mater Sci Eng C.

[2] Tang J, Zhang R, Guo M, Shao L, Liu Y, Zhao Y, Zhang S, Wu Y, Chen C (2018). Nucleosome-inspired nanocarrier obtains encapsulation efficiency enhancement and side effects reduction in chemotherapy by using fullerenol assembled with doxorubicin. Biomaterials.

[3] Romano S, Fonseca N, Simões S, Gonçalves J, Moreira JN (2019). Nucleolin-based targeting strategies for cancer therapy: from targeted drug delivery to cytotoxic ligands. Drug Discov Today.

[4] Bahrami B, Hojjat-Farsangi M, Mohammadi H, Anvari E, Ghalamfarsa G, Yousefi M, Jadidi-Niaragh F (2017). Nanoparticles and targeted drug delivery in cancer therapy. Immunol Lett.

[5] He Z, Zhang Y, Feng N (2020). Cell membrane-coated nanosized active targeted drug delivery systems homing to tumor cells: a review. Mater Sci Eng C.

[6] Taheri-Ledari R, Zhang W, Radmanesh M, Mirmohammadi SS, Maleki A, Cathcart N, Kitaev V (2020). Multi-stimuli nanocomposite therapeutic: docetaxel targeted delivery and synergies in treatment of human breast cancer tumor. Small.

[7] Liu YL, Chen D, Shang P, Yin DC (2019). A review of magnet systems for targeted drug delivery. J Control Release.

[8] Zhang W, Taheri-Ledari R, Hajizadeh Z, Zolfaghari E, Ahghari MR, Maleki A, Hamblin MR, Tian Y (2020). Enhanced activity of vancomycin by encapsulation in hybrid magnetic nanoparticles conjugated to a cell-penetrating peptide. Nanoscale.

[9] Guo J, Gao X, Su L, Xia H, Gu G, Pang Z, Jiang X, Yao L, Chen J, Chen H (2011). Aptamer-functionalized PEG–PLGA nanoparticles for enhanced anti-glioma drug delivery. Biomaterials.

[10] Taheri-Ledari R, Maleki A (2020). Antimicrobial therapeutic enhancement of levofloxacin via conjugation to a cell-penetrating peptide: an efficient sonochemical catalytic process. J Pept Sci.

[11] Cherkasov VR, Mochalova EN, Babenyshev AV, Rozenberg JM, Sokolov IL, Nikitin MP (2020). Antibody-directed metal-organic framework nanoparticles for targeted drug delivery. Acta Biomater.

[12] Majumder J, Taratula O, Minko T (2019). Nanocarrier-based systems for targeted and site specific therapeutic delivery. Adv drug Deliv Rev.

[13] Zhao Z, Ukidve A, Krishnan V, Mitragotri S (2019). Effect of physicochemical and surface properties on in vivo fate of drug nanocarriers. Adv Drug Deliv Rev.

[14] Ayob AZ, Ramasamy TS (2018). Cancer stem cells as key drivers of tumour progression. J Biomed Sci.

[15] McKenzie MD, Ghisi M, Oxley EP, Ngo S, Cimmino L, Esnault C, Liu R, Salmon JM, Bell CC, Ahmed N, Erlichster M (2019). Interconversion between tumorigenic and differentiated states in acute myeloid leukemia. Cell Stem Cell.

[16] Lambert JM, Berkenblit A (2018). Antibody–drug conjugates for cancer treatment. Annu Rev Med.

[17] Shi F, Li M, Wang J, Wu D, Pan M, Guo M, Dou J (2018). Induction of multiple myeloma cancer stem cell apoptosis using conjugated anti-ABCG2 antibody with epirubicin-loaded microbubbles. Stem Cell Res Ther.

[18] Ekladious I, Colson YL, Grinstaff MW (2019). Polymer–drug conjugate therapeutics: advances, insights and prospects. Nat Rev Drug Discov.

[19] Yan J, He W, Yan S, Niu F, Liu T, Ma B, Shao Y, Yan Y, Yang G, Lu W, Du Y (2018). Self-assembled peptide–lanthanide nanoclusters for safe tumor therapy: overcoming and utilizing biological barriers to peptide drug delivery. ACS Nano.

[20] Manivasagan P, Jun SW, Truong NT, Hoang G, Mondal S, Moorthy MS, Kim H, Phan TT, Doan VH, Kim CS, Oh J (2019). A multifunctional near-infrared laser-triggered drug delivery system using folic acid conjugated chitosan oligosaccharide encapsulated gold nanorods for targeted chemo-photothermal therapy. J Mater Chem B.

[21] Kibria G, Ramos EK, Wan Y, Gius DR, Liu H (2018). Exosomes as a drug delivery system in cancer therapy: potential and challenges. Mol Pharm.

[22] Lamichhane N, Udayakumar TS, D’Souza WD, Simone CB, Raghavan SR, Polf J, Mahmood J (2018). Liposomes: clinical applications and potential for image-guided drug delivery. Molecules.

[23] Liyanage PY, Hettiarachchi SD, Zhou Y, Ouhtit A, Seven ES, Oztan CY, Celik E, Leblanc RM (2019). Nanoparticle-mediated targeted drug delivery for breast cancer treatment. Biochim Biophys Acta Rev Cancer.

[24] Nervig CS, Owen SC (2019). Affinity-bound antibody–drug conjugates. Nat Biomed Eng.

[25] Kavand A, Anton N, Vandamme T, Serra CA, Chan-Seng D (2020). Synthesis and functionalization of hyperbranched polymers for targeted drug delivery. J Control Release.

[26] Sur S, Rathore A, Dave V, Reddy KR, Chouhan RS, Sadhu V (2019). Recent developments in functionalized polymer nanoparticles for efficient drug delivery system. Nano-Struct Nano-Objects.

[27] Md S, Bhattmisra SK, Zeeshan F, Shahzad N, Mujtaba MA, Meka VS, Radhakrishnan A, Kesharwani P, Baboota S, Ali J (2018). Nano-carrier enabled drug delivery systems for nose to brain targeting for the treatment of neurodegenerative disorders. J Drug Deliv Sci Technol.

[28] Ju P, Hu J, Li F, Cao Y, Li L, Shi D, Hao Y, Zhang M, He J, Ni P (2018). A biodegradable polyphosphoester-functionalized poly (disulfide) nanocarrier for reduction-triggered intracellular drug delivery. J Mater Chem B.

[29] Parvaz S, Taheri-Ledari R, Esmaeili MS, Rabbani M, Maleki A (2020). A brief survey on the advanced brain drug administration by nanoscale carriers: with a particular focus on AChE reactivators. Life Sci.

[30] Gong Z, Liu X, Dong J, Zhang W, Jiang Y, Zhang J, Feng W, Chen K, Bai J (2019). Transition from vesicles to nanofibres in the enzymatic self-assemblies of an amphiphilic peptide as an antitumour drug carrier. Nanoscale.

[31] Lvov YM, Shchukin DG, Mohwald H, Price RR (2008). Halloysite clay nanotubes for controlled release of protective agents. ACS Nano.

[32] Lvov YM, DeVilliers MM, Fakhrullin RF (2016). The application of halloysite tubule nanoclay in drug delivery. Expert Opin Drug Del.

[33] Kovacevic J, Mladenovic A, Djuris J, Ibric S (2016). Evaluation of powder, solution and suspension layering for the preparation of enteric coated pellets. Eur J Pharm Sci.

[34] Hussan SD, Santanu R, Verma P, Bhandari V (2012). A review on recent advances of enteric coating. IOSR J Pharm.

[35] Shutava TG, Fakhrullin RF, Lvov YM (2014). Spherical and tubule nanocarriers for sustained drug release. Cur Opin Pharmacol.

[36] Hanif M, Jabbar F, Sharif S, Abbas G, Farooq A, Aziz M (2016). Halloysite nanotubes as a new drug-delivery system: a review. Clay Miner.

[37] Price R, Gaber BP, Lvov YR (2001). In-vitro release characteristics of tetracycline HCl, khellin and nicotinamide adenine dineculeotide from halloysite; a cylindrical mineral. J Microencapsul.

[38] Veerabadran NG, Price RR, Lvov YM (2007). Clay nanotubes for encapsulation and sustained release of drugs. NANO.

[39] Kırımlıoğlu GY, Yazan Y (2016). Development, characterization and in vitro release characteristics of rabeprazole sodium in halloysite nanotubes. Eur Int J Sci Technol.

[40] Danyliuk N, Tomaszewska J, Tatarchuk T (2020). Halloysite nanotubes and halloysite-based composites for environmental and biomedical applications. J Mol Liq.

[41] Chauhan VP, Popović Z, Chen O, Cui J, Fukumura D, Bawendi MG, Jain RK (2011). Fluorescent nanorods and nanospheres for real-time in vivo probing of nanoparticle shape-dependent tumor penetration. Angew Chem Int Ed.

[42] Liu Z, Cai W, He L, Nakayama N, Chen K, Sun X, Chen X, Dai H (2007). In vivo biodistribution and highly efficient tumour targeting of carbon nanotubes in mice. Nat Nanotechnol.

[43] Huang X, Teng X, Chen D, Tang F, He J (2010). The effect of the shape of mesoporous silica nanoparticles on cellular uptake and cell function. Biomaterials.

[44] Jain RK (1990). Vascular and interstital barriers to delivery of therapeutic agents in tumors. Cancer Metastasis Rev.

[45] Satish S, Tharmavaram M, Rawtani D (2019). Halloysite nanotubes as a nature’s boon for biomedical applications. Nanobiomedicine.

[46] Dramou P, Fizir M, Taleb A, Itatahine A, Dahiru NS, Mehdi YA, Wei L, Zhang J, He H (2018). Folic acid-conjugated chitosan oligosaccharide-magnetic halloysite nanotubes as a delivery system for camptothecin. Carbohydr Polym.

[47] Hansen EL, Hemmen H, Fonseca DD, Coutant C, Knudsen KD, Plivelic TS, Bonn D, Fossum JO (2012). Swelling transition of a clay induced by heating. Sci Rep.

[48] Santos AC, Ferreira C, Veiga F, Ribeiro AJ, Panchal A, Lvov Y, Agarwal A (2018). Halloysite clay nanotubes for life sciences applications: From drug encapsulation to bioscaffold. Adv Colloid Interface Sci.

[49] Patel S, Jammalamadaka U, Sun L, Tappa K, Mills DK (2016). Sustained release of antibacterial agents from doped halloysite nanotubes. Bio Eng.

[50] Yuan P, Tan D, Annabi-Bergaya F (2015). Properties and applications of halloysite nanotubes: recent research advances and future prospects. Appl Clay Sci.

[51] Li Y, Jin J, Wang D, Lv J, Hou K, Liu Y, Chen C, Tang Z (2018). Coordination-responsive drug release inside gold nanorod@ metal-organic framework core–shell nanostructures for near-infrared-induced synergistic chemo-photothermal therapy. Nano Res.

[52] Abhinayaa R, Jeevitha G, Mangalaraj D, Ponpandian N, Vidhya K, Angayarkanni J (2018). Cytotoxic consequences of Halloysite nanotube/iron oxide nanocomposite and iron oxide nanoparticles upon interaction with bacterial, non-cancerous and cancerous cells. Colloids Surf B.

[53] Huang B, Tian J, Jiang D, Gao Y, Zhang W (2019). NIR-activated “OFF/ON” Photodynamic therapy by a hybrid nanoplatform with upper critical solution temperature block copolymers and gold nanorods. Biomacromol.

[54] Wang J, Zhang Y, Jin N, Mao C, Yang M (2019). Protein-induced gold nanoparticle assembly for improving the photothermal effect in cancer therapy. ACS Appl Mater Inter.

[55] Shafiq-ul-Hassan M, Latifi K, Zhang G, Ullah G, Gillies R, Moros E (2018). Voxel size and gray level normalization of CT radiomic features in lung cancer. Sci Rep.

[56] Saghatchi F, Mohseni-Dargah M, Akbari-Birgani S, Saghatchi S, Kaboudin B (2020). Cancer therapy and imaging through functionalized carbon nanotubes decorated with magnetite and gold nanoparticles as a multimodal tool. Appl biochem biotechnol.

[57] Lin B, Liu J, Wang Y, Yang F, Huang L, Lv R (2020). Enhanced upconversion luminescence-guided synergistic antitumor therapy based on photodynamic therapy and immune checkpoint blockade. Chem Mater.

[58] Nam J, La WG, Hwang S, Ha YS, Park N, Won N, Jung S, Bhang SH, Ma YJ, Cho YM, Jin M (2013). pH-responsive assembly of gold nanoparticles and “spatiotemporally concerted” drug release for synergistic cancer therapy. ACS Nano.

[59] Kang TY, Park K, Kwon SH, Chae WS (2020). Surface-engineered nanoporous gold nanoparticles for light-triggered drug release. Opt Mater.

[60] Das M, Shim KH, An SS, Yi DK (2011). Review on gold nanoparticles and their applications. Toxicol Environ Health Sci.

[61] Yang J, Shen D, Zhou L, Li W, Li X, Yao C, Wang R, El-Toni AM, Zhang F, Zhao D (2013). Spatially confined fabrication of core–shell gold nanocages@ mesoporous silica for near-infrared controlled photothermal drug release. Chem Mater.

[62] Amoli-Diva M, Sadighi-Bonabi R, Pourghazi K (2017). Switchable on/off drug release from gold nanoparticles-grafted dual light-and temperature-responsive hydrogel for controlled drug delivery. Mater Sci Eng C.

[63] Ye B, An C, Zhang Y, Song C, Geng X, Wang J (2018). One-step ball milling preparation of nanoscale CL-20/graphene oxide for significantly reduced particle size and sensitivity. Nanoscale Res Lett.

[64] Allalou S, Kheribet R, Benmounah A (2019). Effects of calcined halloysite nano-clay on the mechanical properties and microstructure of low-clinker cement mortar. Case Stud Constr Mater.

[65] Maleki A, Hajizadeh Z. Acid treatment halloysite nanoclay: Eco-friendly heterogeneous catalyst for the synthesis of pyrrole derivatives. In: Multidisciplinary digital publishing institute proceedings. 2019. 9(1):17.

[66] He C, Hu Y, Yin L, Tang C, Yin C (2010). Effects of particle size and surface charge on cellular uptake and biodistribution of polymeric nanoparticles. Biomaterials.

[67] Deng L, Yuan P, Li D, Du P, Zhou J, Wei Y, Song Y, Liu Y (2019). Effects of calcination and acid treatment on improving benzene adsorption performance of halloysite. Appl Clay Sci.

[68] Hamdi J, Diehl BN, Kilgore K, Lomenzo SA, Trudell ML (2019). Halloysite-catalyzed esterification of Bio-mass derived acids. ACS Omega.

[69] Massaro M, Cavallaro G, Colletti CG, Lazzara G, Milioto S, Noto R, Riela S (2018). Chemical modification of halloysite nanotubes for controlled loading and release. J Mater Chem B.

[70] Fukunaga A, Maeta S, Reema B, Nakakido M, Tsumoto K (2018). Improvement of antibody affinity by introduction of basic amino acid residues into the framework region. Biochem Biophys.

[71] Koniev O, Dovgan I, Renoux B, Ehkirch A, Eberova J, Cianférani S, Kolodych S, Papot S, Wagner A (2018). Reduction–rebridging strategy for the preparation of ADPN-based antibody–drug conjugates. Med Chem Comm.

[72] Lin J, Hu D, Luo Y, Zhong B, Chen Y, Jia Z, Jia D (2019). Functionalized halloysite nanotubes-silica hybrid for enhanced curing and mechanical properties of elastomers. Polymers.

[73] Vega-Vásquez P, Mosier NS, Irudayaraj J (2020). Nanoscale drug delivery systems: From medicine to agriculture. Front Bioeng Biotechnol.

[74] Larm NE, Thon JA, Vazmitsel Y, Atwood JL, Baker GA (2019). Borohydride stabilized gold–silver bimetallic nanocatalysts for highly efficient 4-nitrophenol reduction. Nanoscale Adv.

[75] Hajizadeh Z, Valadi K, Taheri-Ledari R, Maleki A (2020). Convenient Cr(VI) removal from aqueous samples: executed by a promising clay-based catalytic system, magnetized by Fe_3_O_4_ nanoparticles and functionalized with humic acid. ChemistrySelect.

[76] Deng L, Yuan P, Liu D, Du P, Zhou J, Wei Y, Song Y, Liu Y (2019). Effects of calcination and acid treatment on improving benzene adsorption performance of halloysite. Appl Clay Sci.

[77] Garcia-Garcia D, Ferri JM, Ripoll L, Hidalgo M, Lopez-Martinez J, Balart R (2017). Characterization of selectively etched halloysite nanotubes by acid treatment. Appl Surf Sci.

[78] Zhu X, Fan X, Wang Y, Zhai Q, Hu M, Li S, Jiang Y (2021). Amino modified magnetic halloysite nanotube supporting chloroperoxidase immobilization: enhanced stability, reusability, and efficient degradation of pesticide residue in wastewater. Bioprocess Biosyst Eng.

[79] Asempour F, Akbari S, Bai D, Emadzadeh D, Matsuura T, Kruczek B (2018). Improvement of stability and performance of functionalized halloysite nano tubes-based thin film nanocomposite membranes. J Membr Sci.

[80] Bediako EG, Nyankson E, Dodoo-Arhin D, Agyei-Tuffour B, Łukowiec D, Tomiczek B, Yaya A, Efavi JK (2018). Modified halloysite nanoclay as a vehicle for sustained drug delivery. Heliyon..

[81] Long Z, Wu YP, Gao YH, Zhang J, Ou X, He RR, Liu M (2018). In vitro and in vivo toxicity evaluation of halloysite nanotubes. J Mater Chem B.

[82] Saraji M, Tarami M, Mehrafza N (2019). Preparation of a nano-biocomposite film based on halloysite-chitosan as the sorbent for thin film microextraction. Microchem J.

[83] Zhang X, Zhang Z, Xiao L, Ding Z, He J, Lu G, Lu Q, Kaplan DL (2020). Natural nanofiber shuttles for transporting hydrophobic cargo into aqueous solutions. Biomacromol.

[84] Massaro M, Colletti CG, Fiore B, La Parola V, Lazzara G, Guernelli S, Zaccheroni N, Riela S (2019). Gold nanoparticles stabilized by modified halloysite nanotubes for catalytic applications. Appl Organomet Chem.

[85] Kumar P, Paknikar KM, Gajbhiye V (2018). A robust pH-sensitive unimolecular dendritic nanocarrier that enables targeted anti-cancer drug delivery via GLUT transporters. Colloid Surf B.

[86] Persi E, Duran-Frigola M, Damaghi M, Roush WR, Aloy P, Cleveland JL, Gillies RJ, Ruppin E (2018). Systems analysis of intracellular pH vulnerabilities for cancer therapy. Nat Commun.

[87] Fantoni A, Stojkovic V, Carvalho A, Ribeiro AP, Alegria EC (2020). Characterization of AuNPs+ rGO as a functionalized layer for LSPR sensors. Mater Lett.

[88] Eivazzadeh-Keihan R, Chenab KK, Taheri-Ledari R, Mosafer J, Hashemi SM, Mokhtarzadeh A, Maleki A, Hamblin MR (2020). Recent advances in the application of mesoporous silica-based nanomaterials for bone tissue engineering. Mater Sci Eng C.

[89] Guadagno L, Vertuccio L, Naddeo C, Calabrese E, Barra G, Raimondo M, Sorrentino A, Binder WH, Michael P, Rana S (2019). Reversible self-healing carbon-based nanocomposites for structural applications. Polymers.

[90] Sánchez BG, Bort A, Mateos-Gómez PA, Rodríguez-Henche N, Díaz-Laviada I (2019). Combination of the natural product capsaicin and docetaxel synergistically kills human prostate cancer cells through the metabolic regulator AMP-activated kinase. Cancer Cell Int.

[91] Annabi B, Demeule M, Currie JC, Larocque A, Charfi C, Béliveau R (2019). Increasing potency and safety of anticancer drugs through sortilin receptor-mediated cancer therapy: a new targeted approach for the treatment of ovarian cancer. J Clin Oncol.

