# Supplementary material for: Plasmonic photothermal release of docetaxel by gold nanoparticles incorporated onto halloysite nanotubes with conjugated 2D8-E3 antibodies for selective cancer therapy
Source: J Nanobiotechnology. 2021 Aug 11;19:239. doi: 10.1186/s12951-021-00982-6 (PMC8359560; doi:10.1186/s12951-021-00982-6)
Supplement: Supplementary file 1 — Additional file 1: Figure S1. BET analysis: (a) N2 adsorption/desorption, and (b) pore diameter of HNTs. Figure S2. UV–vis spectra for quantification of in vitro DTX release from DTX@HNT/Au-SORT at different experimental conditions. Figure S3. TGA curve of HNTs performed under argon atmosphere. Calculations of cell viability for ables S1 and S2 using the data of Figure S1. Table S1. Optical density data of the wells containing caov-4 cells for in vitro bioassay experiments. Table S2. Optical density data of the wells contating 3T3 cells for in vitro bioassay experiments. Calculation of the ratios of different components in DTX@HNT/Au-SORT based on EDX analysis. [file 12951_2021_982_MOESM1_ESM.docx]

*Additional Information*

**Controlled delivery and release of docetaxel for selective cancer therapy using nanocomposite carriers of halloysite with gold nanoparticle and** **2D8-E3 antibodies**

Reza Taheri-Ledari^1^, Wenjie Zhang^2^, Maral Radmanesh^1^, Ali Maleki*^,1^, Nicole Cathcart^3^, Vladimir Kitaev*^,3^

*^1^ Catalysts and Organic Synthesis Research Laboratory, Department of Chemistry, Iran University of Science and Technology, Tehran 16846-13114, Iran*

*^2^ Department of Nuclear Medicine, West China Hospital, Sichuan University, No. 37, Guoxue Alley, Chengdu 610041, Sichuan Province, P.R. China*

*^3^ Department of Chemistry and Biochemistry, Wilfrid Laurier University, 75 University Ave. W., Waterloo, Ontario, Canada*

*Reza Taheri-Ledari and Wenjie Zhang equally contributed to this work.*

**Corresponding authors. Prof. Ali Maleki: Tel.: +98 21 77240640-50; fax: +98 21 73021584; E-mail address: maleki@iust.ac.ir (A. Maleki). Dr. Vladimir Kitaev: Tel.: +1 519 8840710 x 3643 (Office; E-mail address: vkitaev@wlu.ca (V. Kitaev).*

| Content | Page |
| --- | --- |
| **Figure S1**. BET analysis: (a) N_2_ adsorption/desorption, and (b) pore diameter of HNTs. | S2 |
| **Figure S2**. UV-vis spectra for quantification of *in vitro* DTX release from DTX@HNT/Au-SORT at different experimental conditions. | S3 |
| **Figure S3.** TGA curve of HNTs performed under argon atmosphere. | S4 |
| Calculations of cell viability for ables S1 and S2 using the data of Figure S1. | S5 |
| **Table S1**. Optical density data of the wells containing caov-4 cells for *in vitro* bioassay experiments. | S6 |
| **Table S2**. Optical density data of the wells contating 3T3 cells for *in vitro* bioassay experiments. | S7 |
| Calculation of the ratios of different components in DTX@HNT/Au-SORT based on EDX analysis | S8 |


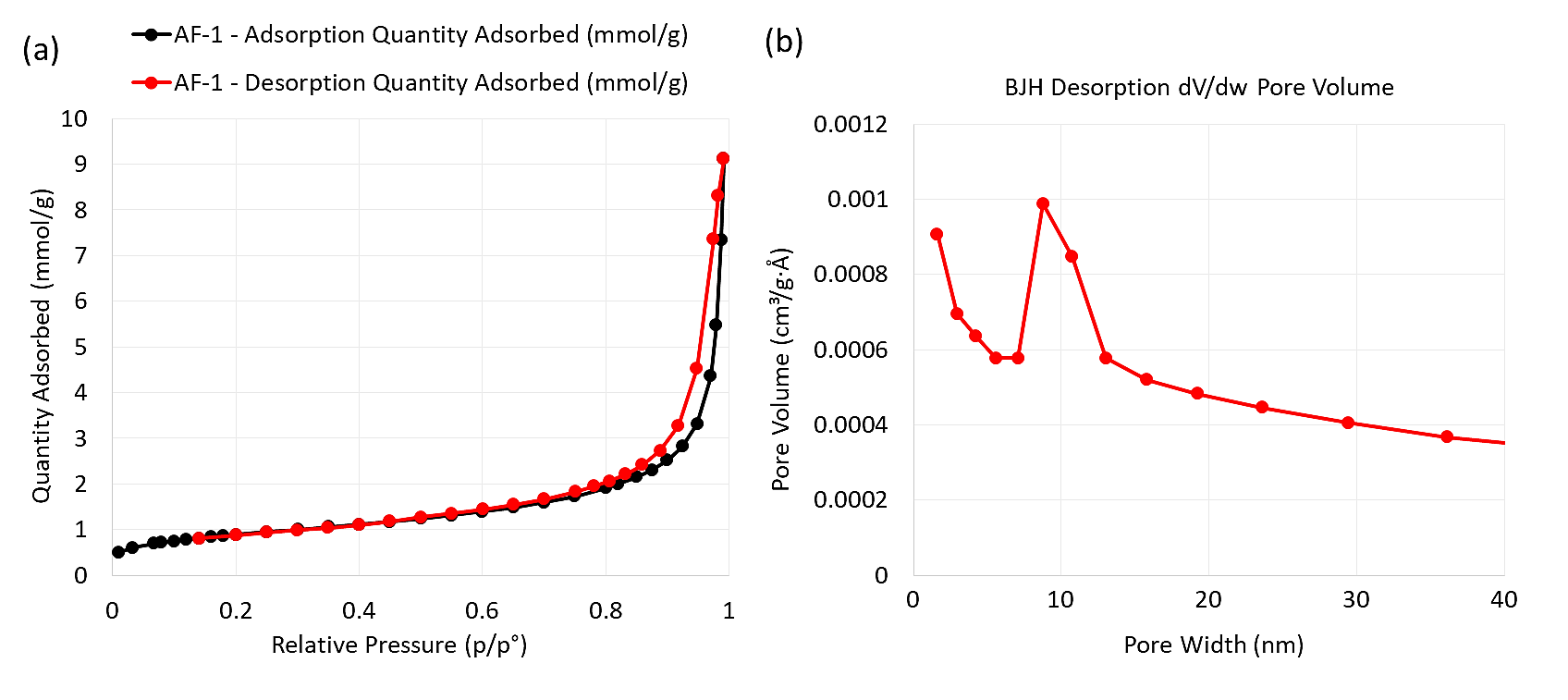


| Pore size characteristics (BJH) | | | BET Surface Area | Sample |
| --- | --- | --- | --- | --- |
| Average pore width | Pore volume | Surface area of pores | 72.3 m²/g | HNTs |
| 9.27 Å | 0.320 cm³/g | 67.7 m²/g |  |  |

**Figure S1**. BET analysis: (a) N_2_ adsorption/desorption, and (b) pore diameter of HNTs.


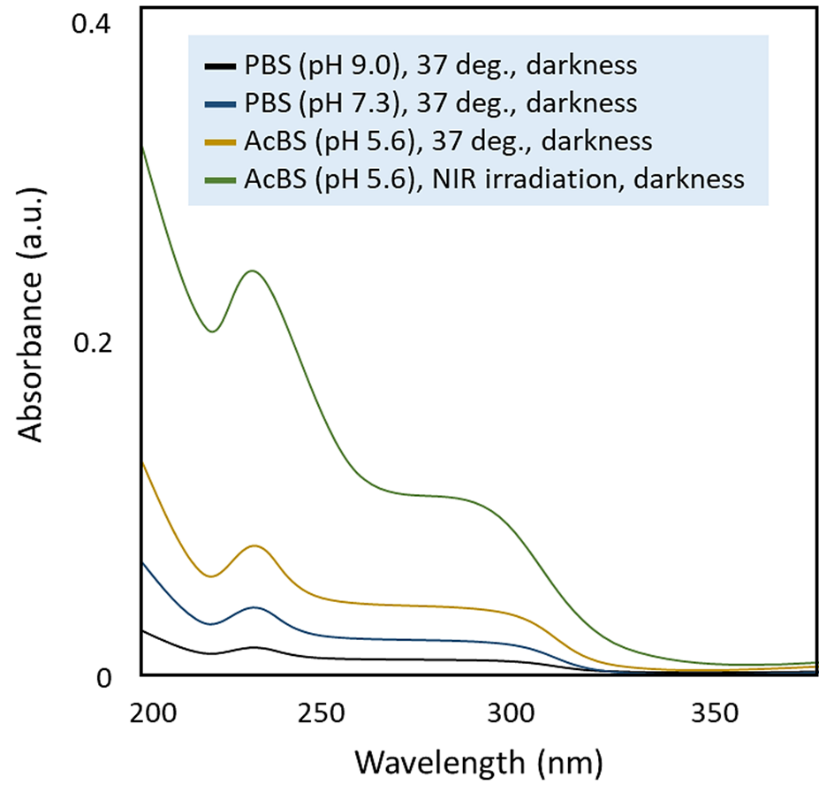


**Figure S2**. UV-vis spectra quantifying *in vitro* DTX release from DTX@HNT/Au-SORT at different experimental conditions.


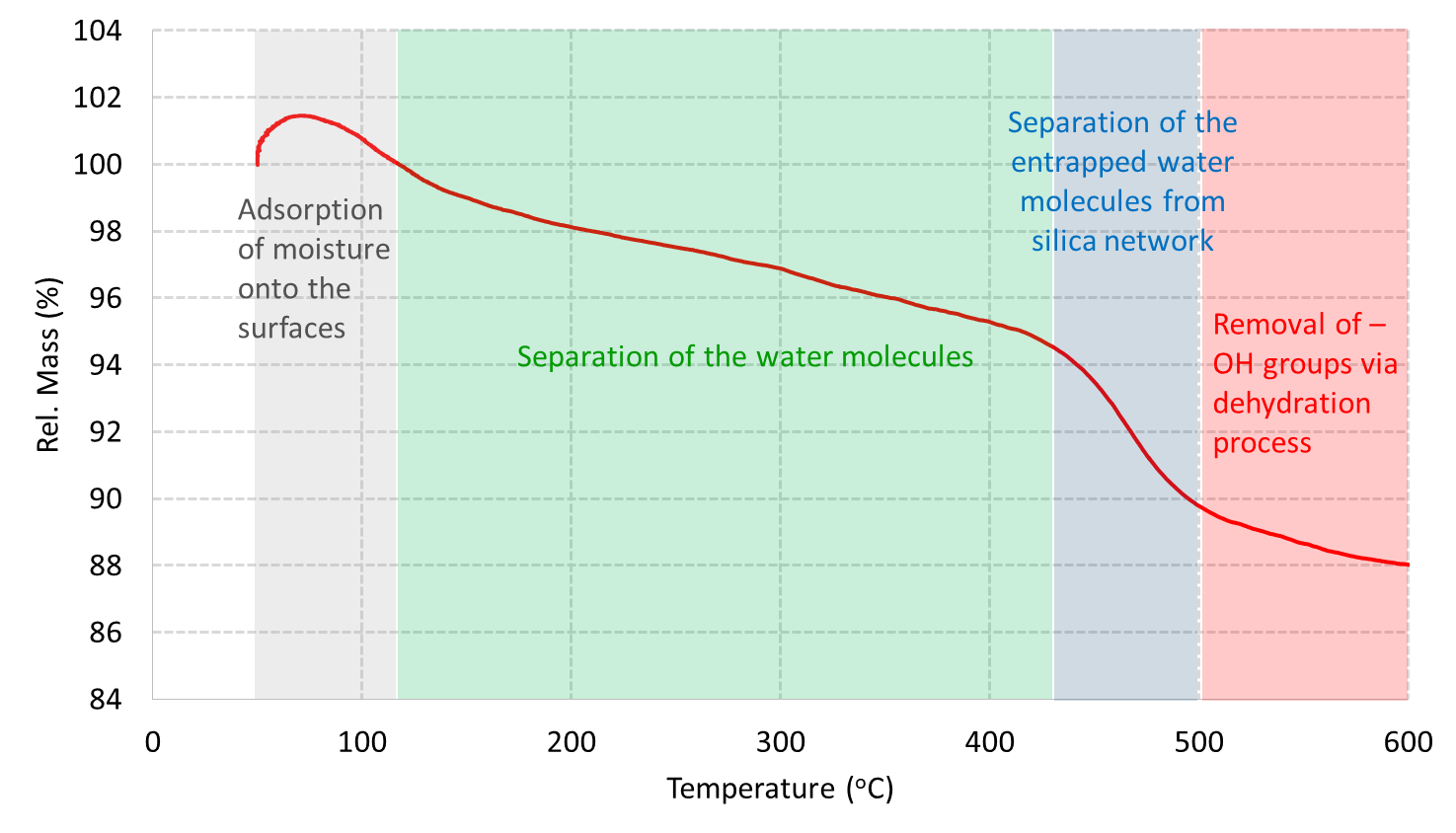
**Figure S3.** Thermal gravimetric anslysis (TGA) curve of HNTs performed under argon atmosphere.

**Cell viability at each condition is calculated using the following equation:**

%CV = - (A/A_0_) × 100

Where, A is UV-vis absorbance of the desired sample, and A_0_ is the UV-vis absorbance of the control at each time.

**Table S1**. Optical density data of the wells containing caov-4 cells for *in vitro* bioassay experiments.

| Conditions | Time (h) | Optical density (a. u.) | Standard deviation | Relative error (%) | Viability (%) |
| --- | --- | --- | --- | --- | --- |
| Caov-4 | 1 | 0.744 | 0.004 | 0.5 | 100 |
| Caov-4 + SORT |  | 0.685 | 0.009 | 1.3 | 92.2 |
| Caov-4 + HNTs |  | 0.736 | 0.008 | 1.0 | 99.0 |
| Caov-4 + DTX |  | 0.675 | 0.003 | 0.4 | 90.7 |
| Caov-4 + AuNPs |  | 0.731 | 0.005 | 0.7 | 98.3 |
| Caov-4 + DTX@HNT |  | 0.729 | 0.006 | 0.8 | 98.0 |
| Caov-4 + DTX@HNT/Au |  | 0.706 | 0.005 | 0.7 | 93.9 |
| Caov-4 + DTX@HNT/Au-SORT (10 μg/mL) |  | 0.691 | 0.004 | 0.6 | 93.0 |
| Caov-4 + DTX@HNT/Au-SORT (30 μg/mL) |  | 0.643 | 0.008 | 1.2 | 86.5 |
| Caov-4 + DTX@HNT/Au-SORT (50 μg/mL) |  | 0.601 | 0.006 | 1.0 | 80.8 |
| Caov-4 | 6 | 0.862 | 0.008 | 0.9 | 100 |
| Caov-4 + SORT |  | 0.760 | 0.005 | 0.6 | 88.2 |
| Caov-4 + HNTs |  | 0.837 | 0.007 | 0.8 | 97.2 |
| Caov-4 + DTX |  | 0.701 | 0.007 | 1.0 | 81.4 |
| Caov-4 + AuNPs |  | 0.827 | 0.006 | 0.7 | 96.0 |
| Caov-4 + DTX@HNT |  | 0.819 | 0.009 | 1.0 | 95.1 |
| Caov-4 + DTX@HNT/Au |  | 0.772 | 0.005 | 0.6 | 89.6 |
| Caov-4 + DTX@HNT/Au-SORT (10 μg/mL) |  | 0.733 | 0.004 | 0.5 | 85.0 |
| Caov-4 + DTX@HNT/Au-SORT (30 μg/mL) |  | 0.650 | 0.007 | 1.0 | 75.5 |
| Caov-4 + DTX@HNT/Au-SORT (50 μg/mL) |  | 0.597 | 0.008 | 1.3 | 69.3 |
| Caov-4 | 24 | 1.102 | 0.004 | 0.4 | 100 |
| Caov-4 + SORT |  | 0.894 | 0.009 | 1.0 | 81.1 |
| Caov-4 + HNTs |  | 1.065 | 0.003 | 0.3 | 96.0 |
| Caov-4 + DTX |  | 0.717 | 0.003 | 0.4 | 65.1 |
| Caov-4 + AuNPs |  | 1.041 | 0.009 | 0.9 | 94.3 |
| Caov-4 + DTX@HNT |  | 1.030 | 0.008 | 0.8 | 93.5 |
| Caov-4 + DTX@HNT/Au |  | 0.793 | 0.007 | 0.9 | 72.0 |
| Caov-4 + DTX@HNT/Au-SORT (10 μg/mL) |  | 0.763 | 0.005 | 0.6 | 69.3 |
| Caov-4 + DTX@HNT/Au-SORT (30 μg/mL) |  | 0.672 | 0.002 | 0.3 | 61.0 |
| Caov-4 + DTX@HNT/Au-SORT (50 μg/mL) |  | 0.445 | 0.005 | 1.1 | 40.4 |
| Caov-4 | 72 | 1.561 | 0.006 | 0.4 | 100 |
| Caov-4 + SORT |  | 1.093 | 0.006 | 0.5 | 70.0 |
| Caov-4 + HNTs |  | 1.482 | 0.006 | 0.4 | 95.0 |
| Caov-4 + DTX |  | 0.596 | 0.008 | 1.3 | 38.2 |
| Caov-4 + AuNPs |  | 1.420 | 0.005 | 0.3 | 91.0 |
| Caov-4 + DTX@HNT |  | 1.397 | 0.005 | 0.3 | 89.5 |
| Caov-4 + DTX@HNT/Au |  | 0.811 | 0.006 | 0.7 | 52.0 |
| Caov-4 + DTX@HNT/Au-SORT (10 μg/mL) |  | 0.610 | 0.008 | 1.3 | 39.1 |
| Caov-4 + DTX@HNT/Au-SORT (30 μg/mL) |  | 0.379 | 0.009 | 2.4 | 24.3 |
| Caov-4 + DTX@HNT/Au-SORT (50 μg/mL) |  | 0.156 | 0.004 | 2.6 | 10.0 |

**Table S2**. Optical density data of the wells contating 3T3 cells for *in vitro* bioassay experiments.

| Conditions | Time (h) | Optical density (a. u.) | Standard deviation | Relative error (%) | Viability (%) |
| --- | --- | --- | --- | --- | --- |
| 3T3 | 1 | 0.711 | 0.003 | 0.4 | 100 |
| 3T3 + SORT |  | 0.712 | 0.009 | 1.3 | 100 |
| 3T3 + HNTs |  | 0.711 | 0.005 | 0.7 | 100 |
| 3T3 + DTX |  | 0.611 | 0.006 | 1.0 | 86.0 |
| 3T3 + AuNPs |  | 0.711 | 0.005 | 0.7 | 100 |
| 3T3 + DTX@HNT |  | 0.968 | 0.008 | 0.8 | 98.2 |
| 3T3 + DTX@HNT/Au |  | 0.696 | 0.002 | 0.3 | 98.0 |
| 3T3 + DTX@HNT/Au-SORT (10 μg/mL) |  | 0.961 | 0.009 | 0.9 | 97.3 |
| 3T3 + DTX@HNT/Au-SORT (30 μg/mL) |  | 0.682 | 0.007 | 1.0 | 96.0 |
| 3T3 + DTX@HNT/Au-SORT (50 μg/mL) |  | 0.661 | 0.007 | 1.0 | 93.1 |
| 3T3 | 6 | 0.825 | 0.006 | 0.7 | 100 |
| 3T3 + SORT |  | 0.828 | 0.005 | 0.6 | 100 |
| 3T3 + HNTs |  | 0.824 | 0.009 | 1.0 | 100 |
| 3T3 + DTX |  | 0.596 | 0.008 | 1.3 | 72.3 |
| 3T3 + AuNPs |  | 0.809 | 0.005 | 0.6 | 98.1 |
| 3T3 + DTX@HNT |  | 0.800 | 0.007 | 0.9 | 97.0 |
| 3T3 + DTX@HNT/Au |  | 0.796 | 0.006 | 0.7 | 96.6 |
| 3T3 + DTX@HNT/Au-SORT (10 μg/mL) |  | 0.801 | 0.004 | 0.5 | 97.0 |
| 3T3 + DTX@HNT/Au-SORT (30 μg/mL) |  | 0.793 | 0.005 | 0.6 | 96.2 |
| 3T3 + DTX@HNT/Au-SORT (50 μg/mL) |  | 0.768 | 0.006 | 0.8 | 93.1 |
| 3T3 | 24 | 0.986 | 0.008 | 0.8 | 100 |
| 3T3 + SORT |  | 0.990 | 0.005 | 0.5 | 100 |
| 3T3 + HNTs |  | 0.967 | 0.003 | 0.3 | 98.1 |
| 3T3 + DTX |  | 0.631 | 0.008 | 1.3 | 64.0 |
| 3T3 + AuNPs |  | 0.959 | 0.004 | 0.4 | 97.3 |
| 3T3 + DTX@HNT |  | 0.951 | 0.005 | 0.5 | 96.5 |
| 3T3 + DTX@HNT/Au |  | 0.950 | 0.005 | 0.5 | 96.2 |
| 3T3 + DTX@HNT/Au-SORT (10 μg/mL) |  | 0.937 | 0.004 | 0.4 | 95.0 |
| 3T3 + DTX@HNT/Au-SORT (30 μg/mL) |  | 0.928 | 0.005 | 0.5 | 94.1 |
| 3T3 + DTX@HNT/Au-SORT (50 μg/mL) |  | 0.873 | 0.006 | 0.7 | 88.6 |
| 3T3 | 72 | 1.442 | 0.008 | 0.5 | 100 |
| 3T3 + SORT |  | 1.445 | 0.009 | 0.6 | 100 |
| 3T3 + HNTs |  | 0.140 | 0.007 | 5.0 | 97.0 |
| 3T3 + DTX |  | 0.644 | 0.007 | 1.0 | 44.7 |
| 3T3 + AuNPs |  | 0.138 | 0.009 | 6.5 | 96.1 |
| 3T3 + DTX@HNT |  | 0.134 | 0.005 | 3.7 | 95.0 |
| 3T3 + DTX@HNT/Au |  | 0.137 | 0.006 | 4.4 | 95.2 |
| 3T3 + DTX@HNT/Au-SORT (10 μg/mL) |  | 0.134 | 0.004 | 3.0 | 93.5 |
| 3T3 + DTX@HNT/Au-SORT (30 μg/mL) |  | 0.131 | 0.008 | 6.1 | 91.2 |
| 3T3 + DTX@HNT/Au-SORT (50 μg/mL) |  | 0.121 | 0.006 | 4.9 | 84.3 |

**Calculations of the ratios of different components in DTX@HNT/Au-SORT based on EDX analysis**

The quantitative values of the elements present in the composite structures are as below:

**HNTs/CPS:**

O: 56.7 wt%

Al: 5.7 wt%

Si: 22.6 wt%

C: 12.7 wt%

Cl: 2.3 wt%

**DTX@HNT/CPS:**

O: 46.1 wt%

Al: 3.6 wt%

Si: 8.9 wt%

C: 40.4 wt%

Cl: 1.0 wt%

**DTX@HNT/Au-SORT:**

C: 42.5 wt%

O: 40.1 wt%

N: 1.1 wt%

Al: 4.0 wt%

Si: 6.3 wt%

S: 1.3 wt%

Au: 4.7 wt%

**A) DTX**

40.4 (±4.6) wt% (carbon-content of DTX@HNT/CPS) – 12.7 (±7.4) wt% (carbon-content of HNT/CPS) = 27.7 (±8.7) wt% (carbon-content of the encapsulated DTX)

27.7 × 807.9 (molecular weight of DTX) / 688 (total weight of carbon in DTC) = 32.5 wt% (loaded-DTX in HNT/CPS)

**B) AuNPs**

From the EDX analysis of DTX@HNT/Au-SORT: the amount of gold is 4.7 wt%. With the atomic weight of the Au is 196.96 g/mol, there is ca. 24 mmol of Au in 100 g of the composite cargo.
